# Targeting cell cycle and apoptotic pathways with newly synthesized diselenide-linked imidazolone analogues with strong CDK6-targeting potential

**DOI:** 10.1039/d5ra10063a

**Published:** 2026-02-04

**Authors:** Marwa Abdel-Motaal, Saad Shaaban, Samia S. Hawas, Asma M. Elsharif, Marwa Sharaky, Fatema S. Alatawi, Mohamed E. Eissa, Arwa Omar Al Khatib, Hany M. Abd El-Lateef, Medhat Asem, Ahmed A. Al-Karmalawy

**Affiliations:** a Department of Chemistry, College of Science, Qassim University Buraidah 51452 Qassim Saudi Arabia; b Organic Chemistry Division, Department of Chemistry, College of Science, Mansoura University Mansoura Egypt; c Department of Chemistry, College of Science, King Faisal University Al-Ahsa 31982 Saudi Arabia sibrahim@kfu.edu.sa; d Department of Pharmaceutical Chemistry, Faculty of Pharmacy, Horus University-Egypt New Damietta 34518 Egypt akarmalawy@horus.edu.eg; e Department of Chemistry, College of Science, Imam Abdulrahman Bin Faisal University Dammam 31441 Saudi Arabia; f Cancer Biology Department, Pharmacology Unit, National Cancer Institute (NCI), Cairo University Cairo Egypt; g Department of Biochemistry, Faculty of Science, University of Tabuk Tabuk Saudi Arabia; h Department of Chemistry, College of Science, Imam Mohammad Ibn Saud Islamic University (IMSIU) Riyadh 11623 Saudi Arabia; i Faculty of Pharmacy, Al-Ahliyya Amman University Amman Jordan; j Department of Civil Engineering, College of Engineering and Information Technology, Onaizah Colleges Qassim 56447 Saudi Arabia; k Department of Pharmaceutical Chemistry, College of Pharmacy, The University of Mashreq Baghdad 10023 Iraq

## Abstract

A novel panel of diselenide-linked imidazolone derivatives was synthesized and biologically profiled, revealing a promising new chemotype with broad-spectrum anticancer activity. Among the series, compounds 6b, 6d, and 6g demonstrated exceptional growth-inhibitory (GI) potency, achieving GI% values of 80.32%, 79.24%, and 86.40%, respectively—substantially outperforming doxorubicin (61.49%). Notably, 6g emerged as the lead candidate, exhibiting robust cytotoxicity across diverse cancer models with IC_50_ values of 6.49 µM (PC3), 6.58 µM (MCF7), 5.38 µM (A549), and 7.25 µM (HCT_116_). Mechanistic studies in A549 cells indicated that 6g simultaneously modulates multiple oncogenic pathways: it markedly downregulated CDK2, CDK4, and CDK6 (1.57–4.12 fold), while upregulating caspase-3, caspase-8, and caspase-9 (1.60–1.64 fold), collectively supporting its dual action on cell-cycle blockade and apoptotic activation. Furthermore, a 1.68-fold reduction in VEGFR-2 expression underscores its additional anti-angiogenic potential. Flow cytometry corroborated these findings, revealing a dramatic S-phase arrest, with the S-phase population rising from 4.61% to 42.09% upon treatment. Several other analogues, including 6d, 6e, 6i, and 6j, also displayed potent cytotoxicity (IC_50_ < 10 µM), highlighting the broader therapeutic relevance of this scaffold. Collectively, these data position 6g as a compelling multi-target anticancer lead that integrates apoptosis induction, cell-cycle regulation, and angiogenesis suppression—supporting its potential for development as a next-generation broad-spectrum anticancer agent.

## Abstract 2

Newly synthesized diselenide-linked imidazolone analogues with great potential towards CDK.

## Graphical abstract

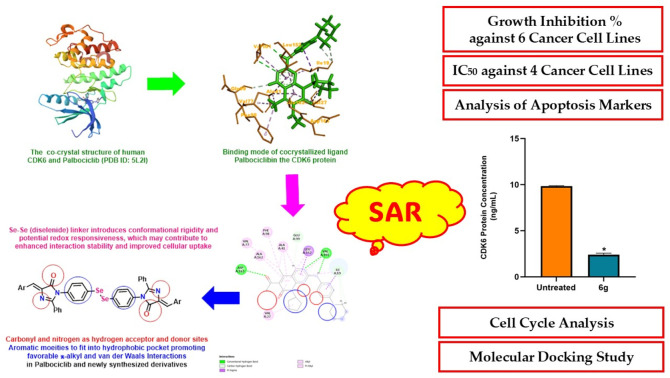

## 1. Introduction

Cancer remains one of the leading causes of morbidity and mortality worldwide, characterized by uncontrolled cellular proliferation, evasion of apoptosis, and the ability to invade surrounding tissues.^1–4^ Among the various types, lung cancer continues to pose a major global health challenge, with non-small cell lung cancer (NSCLC) accounting for nearly 85% of all cases.^5,6^ The A549 cell line, derived from human alveolar adenocarcinoma, is widely used as a representative *in vitro* model for studying NSCLC biology and evaluating potential anticancer agents.^5,7^

A hallmark of malignant transformation is the loss of normal cell-cycle regulation, which allows cancer cells to proliferate indefinitely.^8,9^ The progression of the cell cycle is tightly controlled by a network of cyclins and cyclin-dependent kinases (CDKs).^10,11^ Among these, cyclin-dependent kinase 6 (CDK6) plays a central role in driving the transition from the G1 to S phase by phosphorylating the retinoblastoma (Rb) protein, leading to the release of E2F transcription factors and activation of genes required for DNA synthesis.^12,13^ Overexpression or dysregulation of CDK6 has been documented in several human malignancies, including lung, breast, and hematologic cancers, making it an attractive therapeutic target.^14,15^

Moreover, cell-cycle arrest often triggers apoptosis, a programmed cell-death mechanism essential for eliminating damaged or abnormal cells.^16–18^ Apoptosis involves a cascade of molecular events regulated by pro- and anti-apoptotic proteins such as Bax, Bcl-2, and caspase-3, which serve as key biomarkers for evaluating the apoptotic potential of anticancer agents.^19,20^ In particular, a decrease in Bcl-2 levels coupled with increased Bax expression and activation of caspase-3 is indicative of apoptosis induction.^21,22^

Recent advances in targeted therapy have demonstrated that selective CDK4/6 inhibitors, such as palbociclib, can induce cell-cycle arrest and apoptosis in a variety of tumor models, including A549 cells.^23,24^ This underscores the therapeutic significance of targeting CDK6 to restore normal cell-cycle control and promote apoptotic cell death in cancerous cells.^25,26^ Therefore, designing novel compounds that interfere with CDK6 function represents a promising strategy for the development of effective anticancer agents against NSCLC and other CDK6-driven malignancies.^27^

### 1.1. Rationale design

**Fig. 1:**
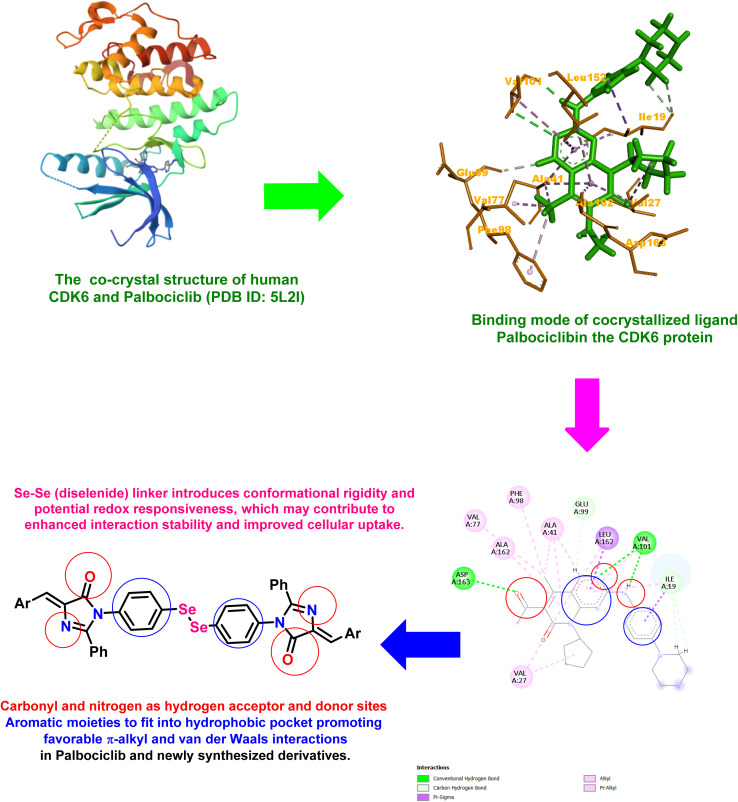
Representation of newly synthesized diselenide-linked imidazolone candidates as potential CDK6 suppression and cell-cycle disruption anticancer agents.

We designed and synthesized a novel series of diselenide-linked imidazolone derivatives that modulate CDK6-associated cell-cycle signaling. The design concept was derived from the co-crystallized ligand palbociclib, which stabilizes the CDK6 receptor (PDB ID: 5L2I) through a network of crucial interactions. Specifically, palbociclib forms two key hinge-region hydrogen bonds with Val101 and Asp163, in addition to a polar contact with Glu99, which collectively anchor the inhibitor within the ATP-binding cleft. Furthermore, multiple hydrophobic residues, including Val27, Val77, Leu152, Phe98, Ala41, and Ala162, surround the aromatic scaffold, forming a nonpolar pocket that enhances ligand accommodation and stability (Fig. 1).

Based on these binding insights, the newly designed scaffold was tailored to preserve the essential pharmacophoric features of palbociclib. The amide carbonyl group and the imidazolone nitrogen within the newly designed compounds can serve as hydrogen bond acceptor and donor sites, respectively, allowing the formation of interactions analogous to those observed between palbociclib and the hinge residues.

The incorporation of a Se–Se (diselenide) linker introduces conformational rigidity and potential redox responsiveness, which may contribute to enhanced interaction stability and improved cellular uptake. Meanwhile, the terminal aromatic or heteroaromatic substituents (Ar) were designed to fit into the hydrophobic pocket, promoting favorable π–alkyl and van der Waals interactions that complement the polar hinge contacts.

Collectively, these structural features were strategically integrated to mirror the ATP-competitive binding mode of palbociclib, while providing new opportunities to optimize structure–activity relationships (SAR) through variation in the aromatic substituents. Hence, this rational design approach combines palbociclib's hinge-binding motif with a diselenide-linked bis-imidazolone framework, aiming to yield novel analogues with improved stability and potent anticancer efficacy against CDK6-driven malignancies.

## 2. Results and discussion

### 2.1. Chemistry

**Scheme 1:**
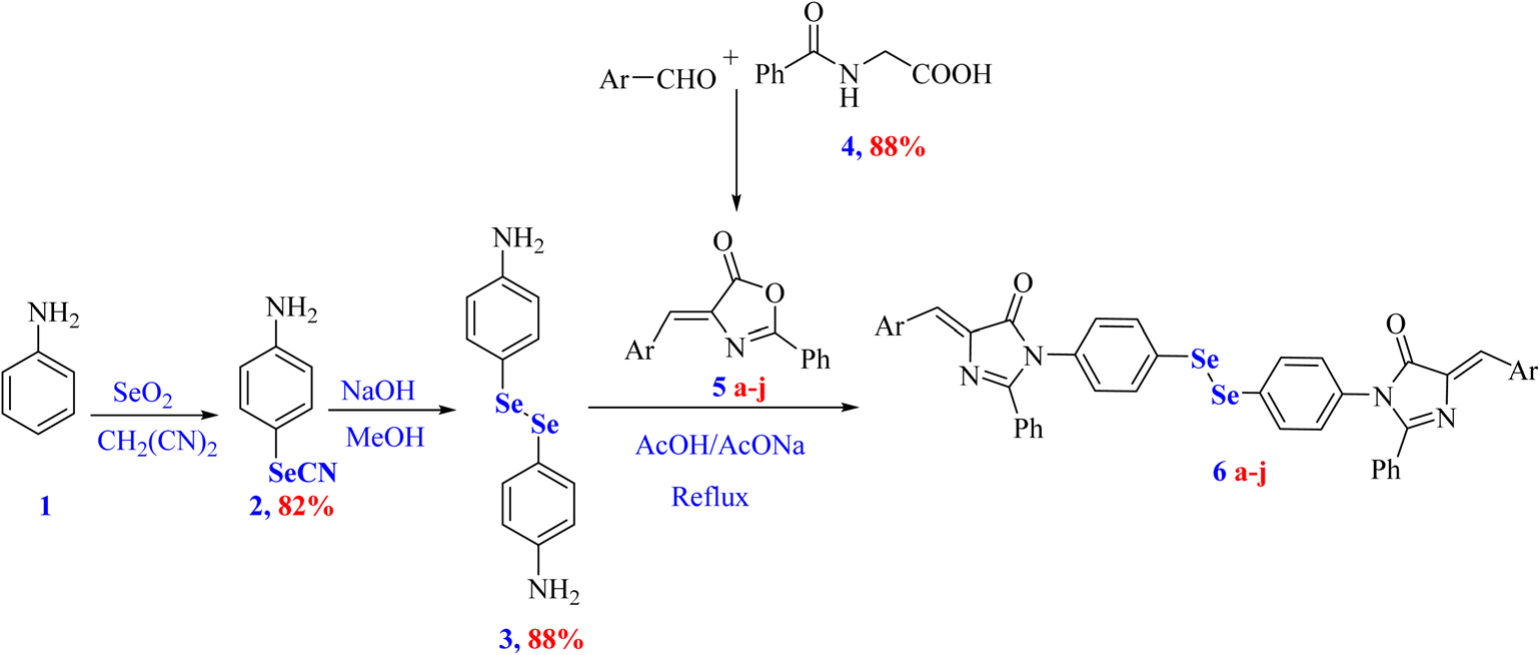
The synthesis of diselenide-based bis-arylidene-4-imidazolone conjugates 6a–j.

Heterocyclic scaffolds remain indispensable in medicinal chemistry due to their broad synthetic versatility and significant therapeutic relevance.^28,29^ Accordingly, our study reports the synthesis of a new series of organoselenium-based imidazolones 6a–j starting from hippuric acid (4), various 4-arylidene oxazolones 6a–j with 4,4′-diselenediyldianiline (3) (Scheme 1). In modern medicinal chemistry and advanced drug discovery, the integration of multiple bioactive pharmacophores into a single molecular framework represents a promising approach.^30,31^ Such a strategy is frequently employed to develop more potent candidates with improved biological efficacy, enhanced selectivity, thereby overcoming limitations related to adverse side effects and drug resistance.

In this context, we envisage designing and synthesizing a series of novel diselenide-based bis-arylidene-4-imidazolone hybrids that incorporate both a 4-imidazolone moiety and a diselenide linkage.^32^ Organic diselenides have garnered considerable attention in recent years due to their promising pharmacological properties, including antioxidant, antibacterial, and anti-inflammatory properties.^32–34^ Despite this promise, further development is often limited by the use of costly and hazardous OSe reagents, demanding reaction conditions, and limited functional-group tolerance.^35,36^ Therefore, there is continuing interest in simple synthetic strategies that employ stable, non-toxic OSe sources compatible with diverse substrates.^33,34,37,38^ In this regard, diaryl diselenides represent valuable precursors for numerous OSe frameworks.^39^ Their high stability facilitates storage and enables efficient optimization of new transformations, and they are commonly used to access a variety of selenium-containing compounds, including organoselenides, selenocyanates, and selenonic acids.^40^

Meanwhile, imidazolones constitute a privileged class of organic heterocycles, found in plants and other living organisms, and they are widely known for their broad spectrum of pharmacological activities.^41^ These scaffolds are also an integral part of many nucleotides (*e.g.*, purine), and several imidazole-based drugs (*e.g.*, fadrozole, dacarbazine, quizartinib, indimitecan, tipifarnib, nilotinib, and ponatinib) are already used for the treatment of a wide range of cancers, including breast cancer, melanoma, lymphomas, and leukemias.^42,43^

Accordingly, the synthesis of diselenide-based bis-arylidene-4-imidazolone conjugates 6a–j is described herein, as illustrated in Scheme 1.

In the initial step, hippuric acid (4) was allowed to react with various aryl aldehydes in the presence of sodium acetate and acetic anhydride to furnish the key intermediate 4-arylidene oxazolones 5a–j. Subsequently, an acetic acid suspension of appropriate 4-arylidene oxazolones 5a–j was refluxed with 4,4′-diselenediyldianiline (3) in the presence of freshly added sodium acetate, afforded the target compounds 6a–j. The yields of the final compounds were high, ranging from 63–95%, as shown in Scheme 1. The most evidence for the formation of final imidazolone targets 6a–j was their IR spectra, which affirms the disappearance of cyclic ester absorption peaks present in oxazolone structures.

The IR spectrum of 6a showed a carbonyl stretching band at 1715 cm^−1^ for the lactam, along with the disappearance of the lactone carbonyl band of the corresponding oxazolone previously observed at 1794 cm^−1^. In addition, the absorption bands of the amino groups of 4,4′-diselenediyldianiline (3) at 3413 and 3404 cm^−1^ were no longer present.

Furthermore, we observed a characteristic band at 1639, 1596 cm^−1^ corresponding to C=N and C=C of arylidene groups, respectively. Similarly, IR spectra of imidazolones 6b–6j exhibit sharp stretching frequencies of the lactam C=O group in the range 1690–1730 cm^−1^, and this differs from the corresponding oxazolones, which show their lactone carbonyl stretching bands in the range (1780–1796 cm^−1^), which confirms the formation of imidazolone moieties.

Moreover, the other bands attributed to some function groups exist, such as characteristic stretching bands at (1651–1632 cm^−1^) for C=N groups, and the exocyclic C=C groups appeared at (1596–1515 cm^−1^). Additionally, the IR spectra show another stretching frequency characterized for each target, such as (827.9 cm^−1^) attributable to C–Cl for 6b, C–NO_2_ appeared at (1484, 1396.9 cm^−1^) for 6c, and (1489.9–1371.3) for 6d. Also, the NH group of the phenothiazine moiety of the compound 6i displayed an absorption band at 3330 cm^−1^. ^1^H-NMR and ^13^C-NMR spectra of the synthesized imidazolones give further support for structural elucidation. Their ^1^H-NMR spectra confirmed that most peaks are located in the aromatic region with complex multiplet signals at *δ* 6.9–8.9 ppm, and the difference between oxazolones 5a–j and imidazolones 6a–j is the integration of the signals in the aromatic region beside the presence of singlet signals in the range of *δ* 8.2–9.1 ppm due to the exocyclic –CH=C protons, which confirms the formation of the target compounds by condensation of the oxazolones with the 4,4′-diselenediyldianiline supported with the disappearance of the –NH_2_ proton signals of the diselenide compound 3. On the other hand, the ^1^H-NMR spectrum of the formed imidazolones showed characteristic signals according to the condensed aromatic aldehyde fragment. Trimethoxy protons in the case of compound 6e were observed as two singlet signals at *δ* 3.7 ppm (2-OCH_3_) and 3.8 ppm (4-OCH_3_), which revealed the formation of the bis-arylidene–imidazolone derivatives. Further, we detected a singlet signal at *δ* 7.2 ppm for compound 6f belongs to (2O–CH_2_–O) protons. The two NH protons of the phenothiazine fragment for compound 6i were observed at *δ* 9.6 ppm. Besides, a singlet signal due to two acetyl protons for compound 6j was displayed at *δ* 2.09 ppm, which indicates the occurrence of acetylation on the –NH group of the indole fragment after imidazolone cyclization. ^13^C-NMR spectra of the formed structures proved, besides the aromatic signals' presence of carbonyl amide for all targets, approaching *δ* 169 ppm. Additionally, aliphatic carbons of some compounds were observed, including two characteristic signals at *δ* 65.3 ppm due to the four methoxy carbons and 60.7 ppm due to the two methoxy carbons for compound 6e. Also, it is observed that there is a specific signal at *δ* 99.06 ppm owing to the methylene carbon of the dioxolo group for compound 6f. Finally, the methyl carbon of the acetyl group for compound 6i was observed at *δ* 24.5 ppm, which gave good evidence for the acetylation of the –NH group (Table 1).

**Table 1:** Novel diselenide-based bis-arylidene-4-imidazolone hybrids 6a–j

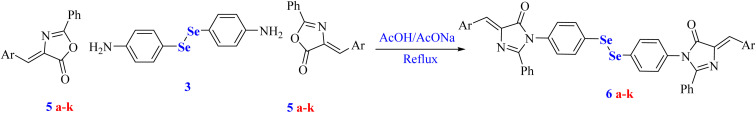				
Analogue	Ar	MP (°C)	Colour	Yield %
6a	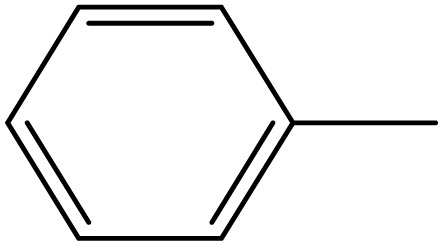	98–99	Bage	88
6b	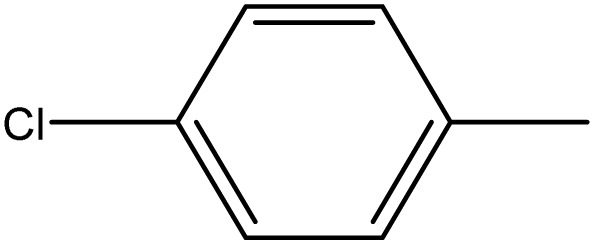	230	Yellow	60
6c	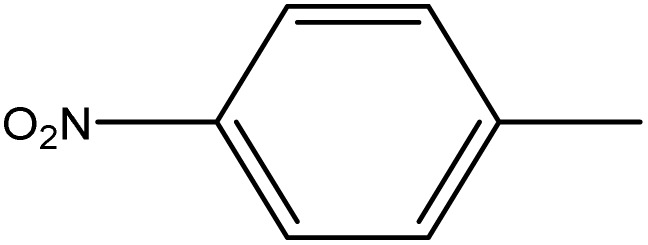	249–250	Yellow	93
6d	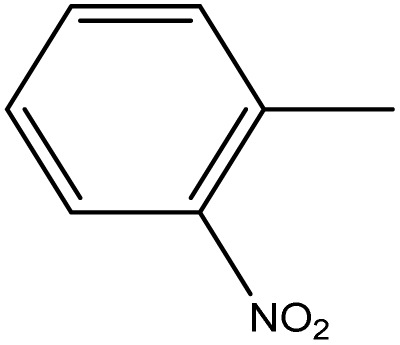	124–125	Deep yellow	85
6e	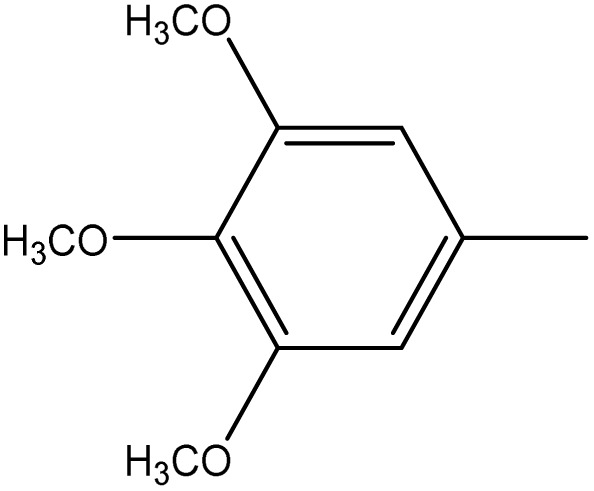	220–221	Orange	83
6f	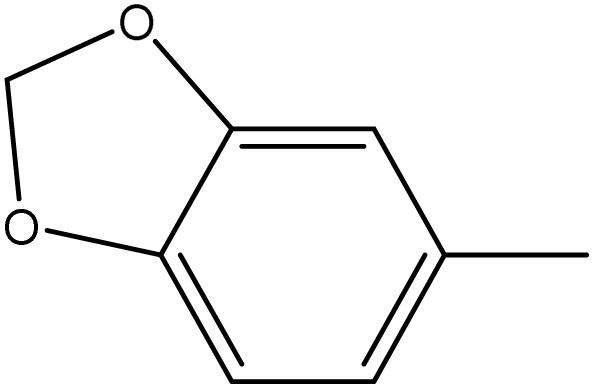	170–171	Yellow	95
6g	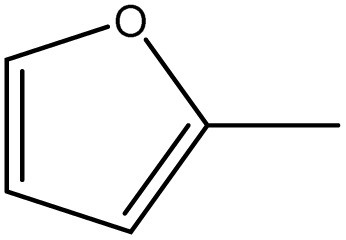	114–115	Yellow	75
6h	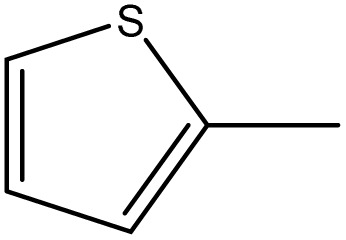	>300	Deep yellow	92
6i	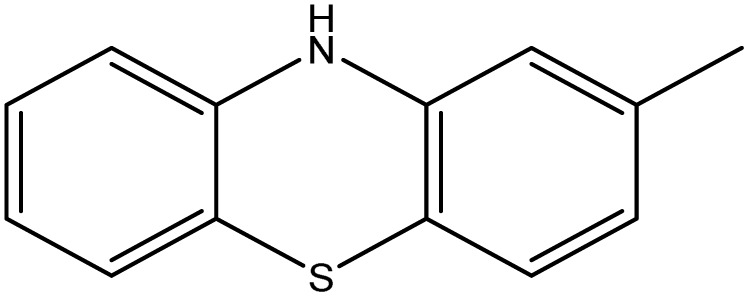	198–199	Deep blue	65
6j	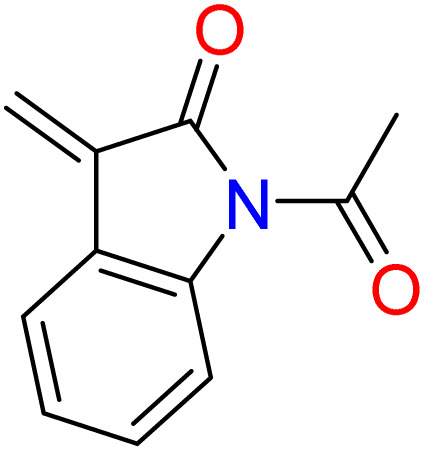	124–125	Brown	60

### 2.2. Biological assays

#### 2.2.1. Cellular growth inhibition percentage (GI%) against different cancerous cell lines

The newly synthesized organoselenium–imidazolone hybrids (6a–j) were tested against six different cancer cell lines to evaluate their growth inhibition potential (GI%). These include colorectal carcinoma (HCT_116_), human breast adenocarcinoma (MCF7), hepatocellular carcinoma (HuH7), prostate carcinoma (PC3), lung adenocarcinoma (A549), and osteosarcoma (MG63). Table 2 shows the GI% values of the newly synthesized compounds against doxorubicin (DOX), used as a reference drug.

**Table 2:** Growth inhibition percentage (GI%) of novel organoselenium derivatives against six cancer cell lines

Cell line/comp.	6a	6b	6c	6d	6e	6f	6g	6h	6i	6j	DOX	Average
HCT_116_	73.06	86.12	71.14	74.27	73.09	48.00	81.57	74.41	81.13	78.15	64.36	73.72
MCF7	73.69	81.26	68.21	80.33	76.69	48.23	91.10	72.07	71.4	76.73	58.65	73.31
HuH7	64.97	78.91	74.86	64.81	59.31	9.91	83.17	72.62	76.04	60.22	68.00	65.16
PC3	90.45	80.39	83.55	87.56	87.95	56.03	89.58	83.41	90.51	85.11	68.92	82.13
A549	89.93	89.02	85.17	90.10	83.46	52.02	92.11	88.31	90.77	87.56	39.14	81.18
MG63	68.83	66.19	73.47	78.34	47.34	9.84	80.84	60.16	61.04	79.59	69.86	63.23
Average	76.82	80.32	76.07	79.24	71.31	37.34	86.40	75.16	78.48	77.89	61.49	
HSF	69.98	74.54	61.71	76.41	61.65	5.93	74.42	68.00	68.22	67.49	29.57	59.81

#### 2.2.2. Structure–activity relationships (SAR)

From Table 2, it is clear that compounds 6b, 6d, and 6g demonstrated the highest average GI% values (80.32%, 79.24%, and 86.40%, respectively), markedly surpassing the reference drug doxorubicin (57.54%). These compounds showed potent inhibition across multiple cancer cell lines, particularly A549, PC3, and HCT_116_, highlighting their strong cytotoxic potential.

A detailed analysis of the structural features of the synthesized diselenide–imidazolone derivatives revealed that their cytotoxic activity is strongly governed by both the electronic nature and steric environment of the substituents on the two aromatic rings attached to the imidazolone core. Interestingly, both electron-donating and electron-withdrawing groups contributed to pronounced cytotoxicity, albeit likely through distinct mechanisms. Electron-donating substituents tend to enhance lipophilicity and facilitate cell membrane permeation, whereas electron-withdrawing substituents increase the electrophilicity of the Se–C bond, thereby intensifying oxidative stress or enabling enzyme inhibition in cancer cells.

Among all the tested analogues, compound 6g, featuring two furan rings symmetrically positioned on both sides of the molecule, emerged as the most potent member of the series. This unique configuration may favor π–π stacking interactions and optimize binding affinity toward intracellular targets, leading to strong inhibition across multiple cancer cell lines.

Compounds containing electron-withdrawing substituents, such as –Cl or –NO_2_ (notably in 6b, 6c, and 6d), also demonstrated remarkable inhibitory effects, particularly against HCT_116_, MCF7, and A549 cell lines. The increased electrophilicity imparted by these groups may facilitate redox modulation and trigger oxidative damage in malignant cells, accounting for their strong cytotoxic responses.

In contrast, unsubstituted derivatives such as 6a and 6h exhibited moderate activity. This reduction in activity likely arises from bulky structural features that restrict optimal target interaction and impede cell penetration.

**Fig. 2:**
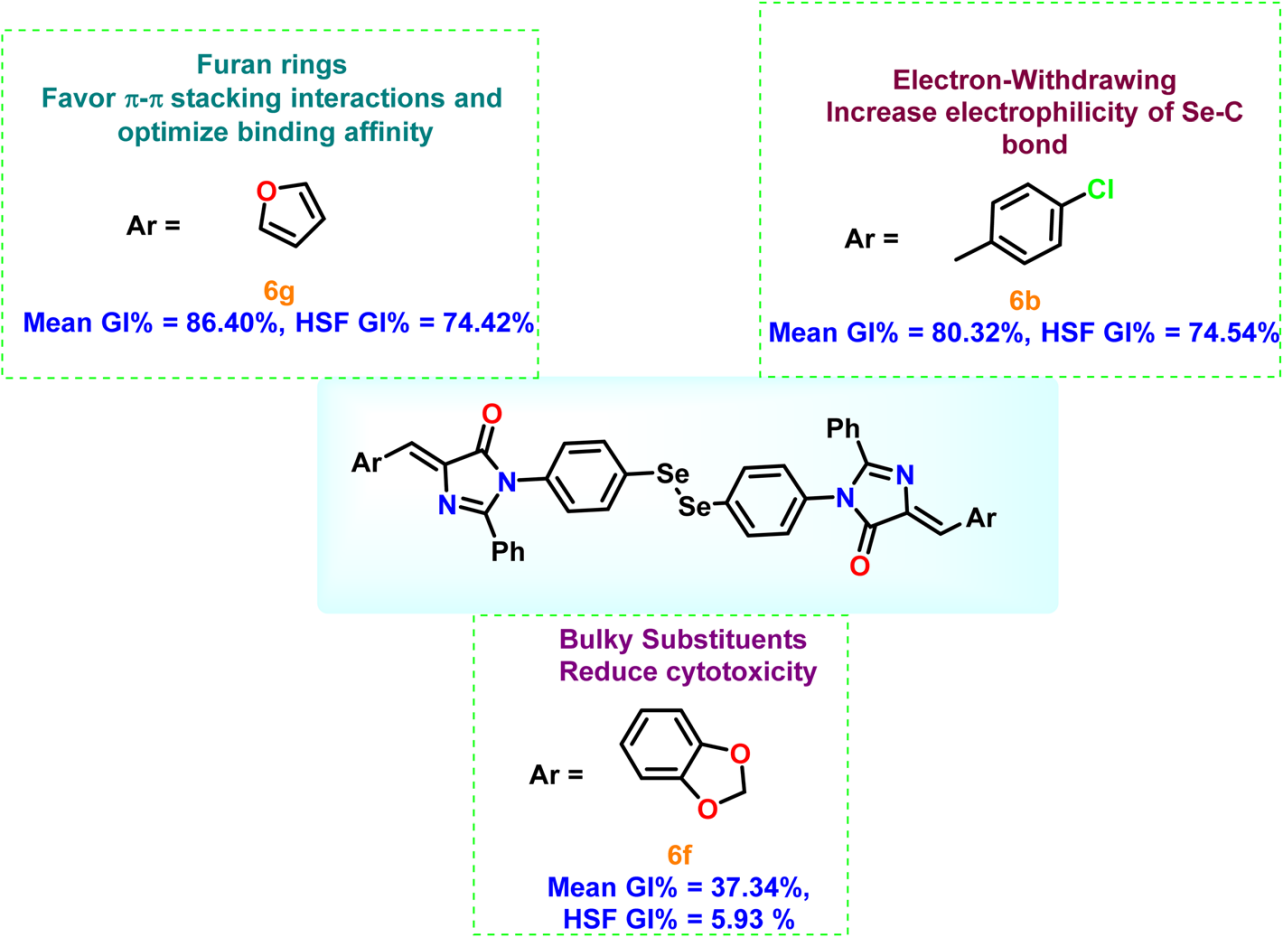
Structure–activity relationship of the newly synthesized diselenide-linked imidazolone derivatives.

Notably, 6g displayed moderate cytotoxicity against normal HSF cells (GI% = 74.42%), indicating some selectivity toward cancer cells. Similarly, 6b and 6d exhibited GI% values of 74.54% and 76.41% in HSF cells, respectively, suggesting lower selectivity despite strong cancer cell inhibition. In contrast, compounds 6a, 6c, 6e, and 6h–6j showed moderate cytotoxicity in both cancer and normal cells, while the sterically hindered 6f displayed the lowest activity (average GI% = 37.34% in cancer cells and 5.93% in HSF cells), reflecting reduced cell penetration and target interaction. The structure–activity relationship is illustrated in Fig. 2.

Selenium-containing compounds are known to exert cytotoxicity through redox-related mechanisms, including induction of reactive oxygen species (ROS). While direct ROS measurements were not performed in this study, the observed cytotoxicity patterns suggest that redox modulation may contribute to the antiproliferative effects of the synthesized diselenide–imidazolone derivatives. To evaluate selectivity, we calculated a selectivity index (SI = GI% in normal HSF/average GI% in cancer cells) for each compound (Table 3). Compound 6g demonstrated high potency across cancer cell lines (average GI% = 86.40%) with moderate normal cell toxicity (GI% HSF = 74.42%, SI = 0.86), indicating a favorable therapeutic window. In contrast, compound 6f exhibited very low activity in both cancer and normal cells (SI = 0.16), while DOX showed lower potency with a higher relative selectivity (SI = 0.48).

**Table 3:** Average growth inhibition (GI%) of diselenide–imidazolone derivatives in cancer cell lines and their GI% in normal HSF cells, and calculated selectivity index (SI = GI% HSF/average GI% cancer)

Compound	Average GI% cancer	GI% HSF	aSI (HSF/cancer)
6a	76.82	69.98	0.91
6b	80.32	74.54	0.93
6c	76.07	61.71	0.81
6d	79.24	76.41	0.96
6e	71.31	61.65	0.86
6f	37.34	5.93	0.16
6g	86.40	74.42	0.86
6h	75.16	68.00	0.90
6i	78.48	68.22	0.87
6j	77.89	67.49	0.87
DOX	61.49	29.57	0.48

a SI values closer to 1 indicate lower selectivity, whereas lower SI values indicate higher selectivity toward cancer cells.

Overall, these results suggest that structural modifications can modulate both potency and selectivity. Importantly, despite the known redox-related toxicity of selenium compounds, the moderate cytotoxicity toward normal HSF cells observed for the most active analogues, particularly 6g, supports their potential for further development as anticancer agents, while highlighting the need for careful dose optimization and safety evaluation in future studies.

#### 2.2.3. Evaluation of cytotoxic inhibitory concentration 50 (IC_50_) against PC3, MCF7, A549, and HCT_116_ cells

**Fig. 3:**
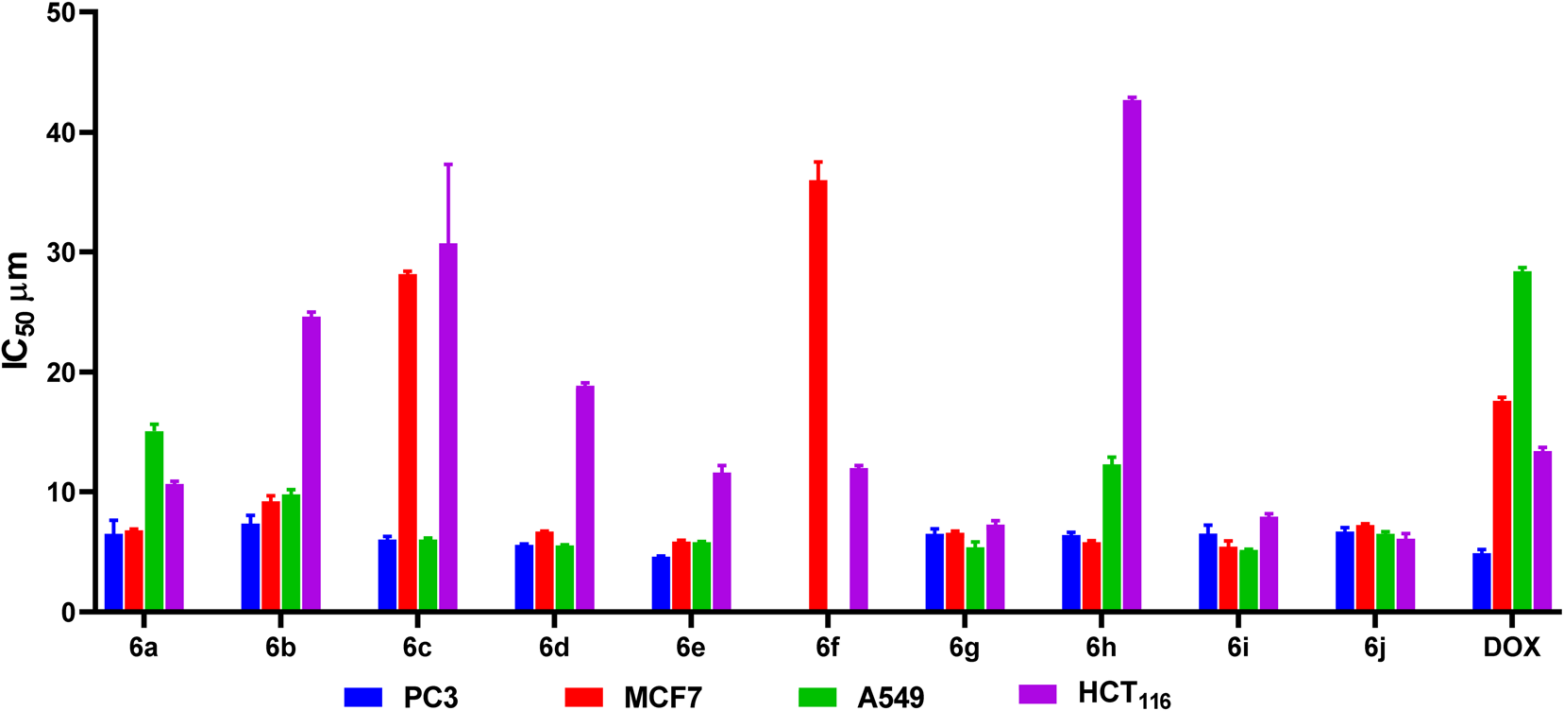
Assessment of the inspected compounds 6a–j cytotoxic inhibitory concentration 50 (IC_50_) against the cancer cell lines: PC3, MCF7, A549, and HCT_116_ using DOX as a reference.

The cytotoxic potential of compounds 6a–j was evaluated against PC3, MCF7, A549, and HCT_116_ cell lines using the sulforhodamine B (SRB) assay^44^ at concentrations of 12.5, 25, 50, and 100 µM equivalents (Fig. 3).

Among the tested compounds, 6g emerged as one of the most potent derivatives, displaying the highest average GI% (86.40%) and strong cytotoxic activity with IC_50_ values of 6.49 µM (PC3), 6.58 µM (MCF7), 5.38 µM (A549), and 7.25 µM (HCT_116_). Likewise, 6e exhibited the lowest IC_50_ values overall (4.57, 5.83, 5.80, and 11.63 µM against PC3, MCF7, A549, and HCT_116_, respectively), indicating high potency comparable to doxorubicin (4.88, 17.59, 28.38, and 13.40 µM).

Other derivatives, including 6d (5.56, 6.69, 5.52, and 18.86 µM), 6i (6.50, 5.42, 5.14, and 7.93 µM), and 6j (6.68, 7.24, 6.49, and 6.08 µM), also demonstrated promising cytotoxic profiles with IC_50_ values mostly in the low micromolar range.

In contrast, 6c and 6h showed noticeably weaker activity, particularly against HCT_116_ cells (30.72 and 42.64 µM, respectively).

Overall, compounds 6d, 6e, 6g, 6i, and 6j can be considered the most potent members of this series, exhibiting IC_50_ values below 10 µM in several cancer cell lines, suggesting their strong antiproliferative potential.

#### 2.2.4. Protein expression of apoptosis and cell cycle-related proteins in A549 cells treated with 6g

The effects of compound 6g on key regulatory proteins involved in apoptosis, cell cycle control, and angiogenesis were examined in A549 lung cancer cells. Treatment with 6g significantly altered the expression of these proteins, supporting its potential multi-target anticancer mechanism.

Specifically, cell cycle-related proteins CDK2, CDK4, and CDK6 were downregulated with fold changes of 1.57, 1.76, and 4.12, respectively, indicating partial suppression of cell cycle progression. Pro-apoptotic proteins (caspase-3, caspase-8, and caspase-9) were modestly upregulated (fold changes 1.60, 1.62, and 1.64), respectively, suggesting activation of both intrinsic and extrinsic apoptotic pathways. VEGFR-2 expression decreased by 1.68-fold, which may indicate a potential impact on angiogenic signaling; however, functional assays such as tube formation or migration would be required to confirm anti-angiogenic activity.^45,46^

**Fig. 4:**
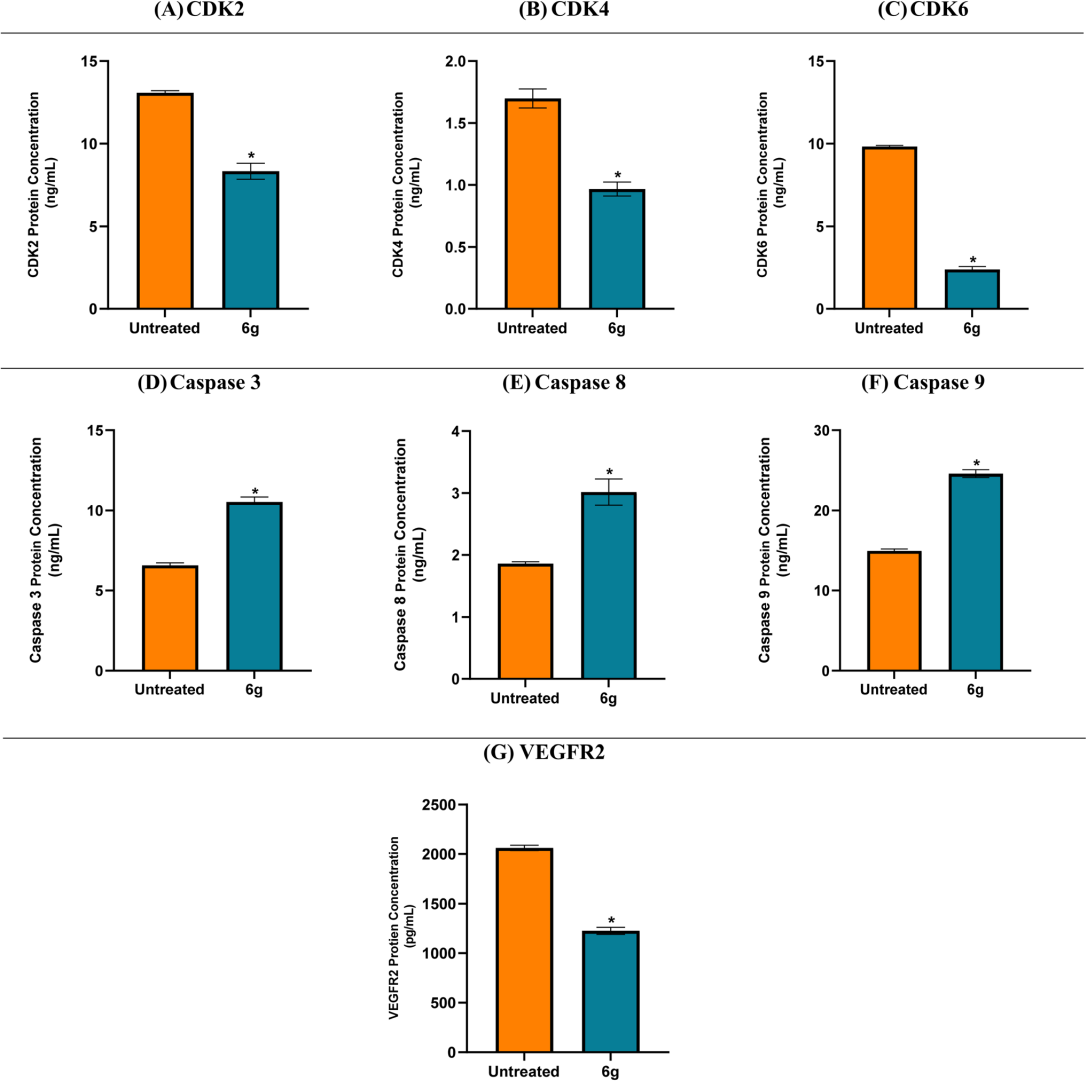
CDK2 (A), CDK4 (B), and CDK6 (C), Caspase 3 (D), Caspase 8 (E), and Caspase 9 (F), and VEGFR2 (G) protein expression levels for compound 6g in the treated and untreated A549 cancer cell line.

Overall, these findings indicate that 6g may modulate multiple regulatory pathways, including apoptosis, cell cycle progression, and angiogenesis, although the observed effects are moderate and warrant further functional validation to establish causal relationships (Fig. 4).

#### 2.2.5. Evaluation of cell cycle arrest for 6g in the A549

**Fig. 5:**
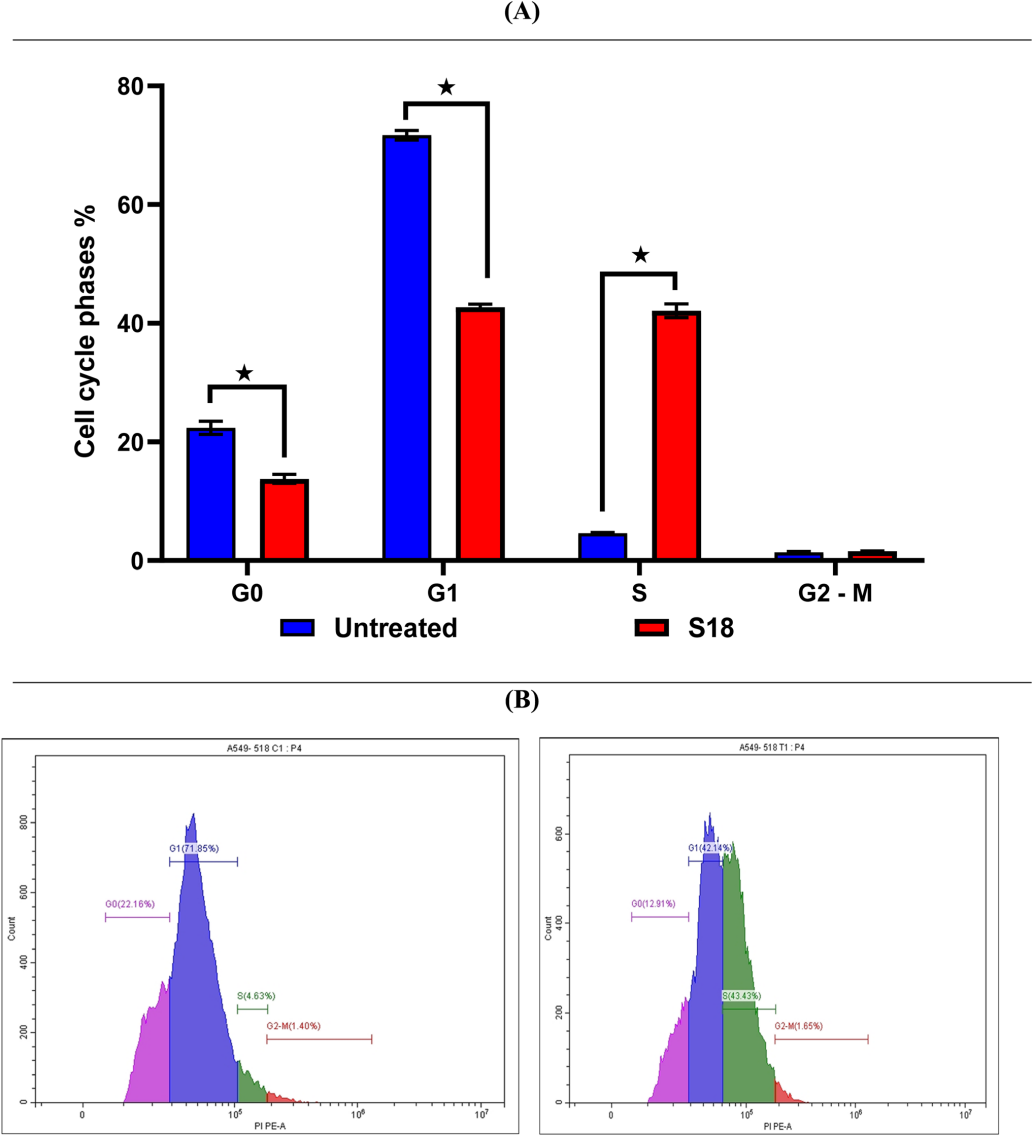
(A) Cell cycle analysis of treated & untreated A549 cells with compound 6g. (B) Histograms of A549 treated with compound 6g (left side) *vs.* control (right side).

To further elucidate the anticancer activity of compound 6g, a flow cytometric analysis was conducted to evaluate its effect on the cell cycle distribution of A549 lung cancer cells.^47–49^ The analysis demonstrated a distinct alteration in cell cycle progression following 6g treatment. Specifically, the proportion of cells in the G0 phase decreased from an average of 22.37% in untreated cells to 13.78% in treated cells, while the G1 phase population declined from 71.68% to 42.69%. In contrast, the percentage of cells in the S phase markedly increased from 4.61% to 42.09%, indicating a pronounced S-phase arrest. The G2/M phase remained relatively stable, showing only a slight change (1.39% in untreated *vs.* 1.6% in treated cells) as in Fig. 5.

While these results indicate S-phase accumulation, it likely reflects replication stress or downstream cell cycle regulation rather than direct CDK6 inhibition, as CDK4/6 classically regulate the G1–S transition. Selenium-containing compounds, including diselenides, are known to induce oxidative stress and DNA damage, which can converge to produce S-phase arrest.^50–52^ Therefore, the observed effect supports the conclusion that 6g exerts multifactorial cytotoxic effects, rather than exclusive CDK4/6 inhibition. The experiment was performed three times to ensure reproducibility, and the corresponding histograms are provided in the SI.

### 2.3. Molecular docking

A molecular docking study was conducted to investigate the apoptotic potential of analogue 6g against the CDK6 target receptor (PDB ID: 5L2I). Compound 6g was selected for molecular docking studies because it exhibited the highest GI% across cancer cell lines and the most favorable selectivity profile toward normal HSF cells among all tested analogues. Accordingly, 6g served as a representative lead compound to explore potential molecular interactions with the CDK6 target receptor.

**Fig. 6:**
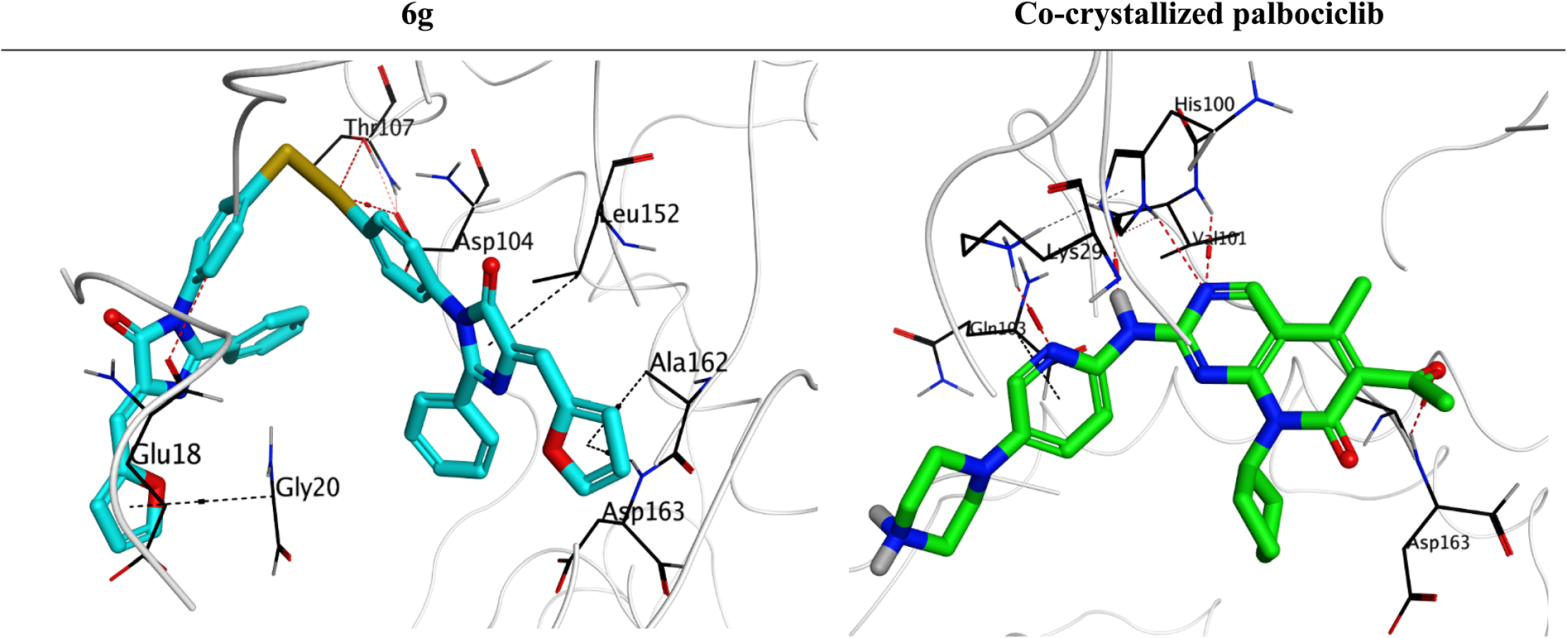
3D binding interactions of analogue 6g and the co-crystallized palbociclib within the active site of CDK6 receptor (PDB ID: 5L2I).

Compound 6g achieved a binding score of −9.55 kcal mol^−1^ (RMSD = 1.69 Å), which was very promising and comparable to that of the CDK6 co-crystal palbociclib inhibitor (binding score = −10.53 kcal mol^−1^ and RMSD = 1.71 Å). Analogue 6g got stabilized inside the binding pocket of the CDK6 receptor through the formation of three hydrogen bonds with Thr107, Asp104, and Glu18. Also, it formed four pi–hydrogen interactions with Asp163, Ala162, Leu152, and Gly20. On the other side, the CDK6 co-crystal palbociclib inhibitor showed five hydrogen bonds with Asp163, Val101 (2), His100, and Lys29. Besides, it formed one pi–hydrogen interaction with Gln103 (Fig. 6). Accordingly, the docking study results were in accordance with the previously discussed *in vitro* ones, indicating a potential suppression activity for compound 6g towards the CDK6 target receptor. While these docking results suggest that 6g could interact with CDK6, it is important to note that docking alone cannot confirm target engagement or selectivity. Therefore, the observed *in vitro* cytotoxicity may arise from CDK6 inhibition or from other nonspecific effects. These results serve as a computational prediction supporting further experimental validation of 6g as a potential CDK6 inhibitor.

## 3. Conclusion

The present study highlights the strong therapeutic promise of the newly synthesized diselenide-linked imidazolone derivatives, several of which demonstrated superior cytotoxic performance compared to DOX. Among the evaluated analogues, 6b, 6d, and 6g were the most active, exhibiting average GI% values of 80.32%, 79.24%, and 86.40%, respectively, underscoring their broad anticancer potential. Compound 6g, in particular, emerged as a standout candidate, displaying potent activity across diverse cancer cell lines with IC_50_ values of 6.49 µM (PC3), 6.58 µM (MCF7), 5.38 µM (A549), and 7.25 µM (HCT_116_). Mechanistic investigations revealed that 6g profoundly disrupts multiple oncogenic signaling axes. It significantly downregulated CDK2, CDK4, and CDK6 (1.57-, 1.76-, and 4.12-fold decreases) as well as VEGFR-2 (1.68-fold decrease), consistent with both cell cycle suppression and anti-angiogenic action. Simultaneously, caspase-3, caspase-8, and caspase-9 were upregulated (1.60–1.64-fold), verifying activation of apoptotic pathways. Flow cytometric analysis further confirmed these molecular events, revealing a striking S-phase arrest, where the S-phase fraction increased from 4.61% to 42.09%, accompanied by marked reductions in G0 and G1 populations (13.78% and 42.69%, respectively). Collectively, these findings establish that the diselenide-linked imidazolone scaffold—particularly compound 6g—acts through a coordinated, multi-target mechanism involving apoptosis induction, cell cycle blockade, and inhibition of angiogenesis. Such a profile positions this chemotype as a promising platform for further optimization and development of next-generation anticancer agents.

## 4. Materials and methods

### 4.1. Chemistry

#### 4.1.1. General

The reagents (such as hippuric acid, aromatic aldehydes, acetic anhydride, sodium acetate, aniline, and SeO_2_), solvents, and thin-layer chromatography (TLC) aluminum plates coated with silica gel (60 F254, Merck) utilized in the synthesis of the targets were purchased from Sigma-Aldrich. TLC was employed to monitor the completion of the reaction and to confirm the purity of the products. Visualization of the spots at TLC was achieved under UV light at 254 nm and 365 nm. Measuring the melting point °C *via* the Stuart SMP30 apparatus, and no corrections were applied. NMR spectra of the synthesized targets were assessed using a Bruker NMR spectrometer (at Faculty of Pharmacy, Mansoura University in Mansoura, Egypt) operating at frequencies of 400 MHz (^1^H-NMR) and 100 MHz (^13^C-NMR). TMS (tetramethylsilane) was used as reference material, and the synthesized compounds were dissolved in DMSO-d6 or in TFA (*d*-trifluoroacetic acid) in *δ* (ppm). Measurement of IR spectra *via* Shimadzu Model 8000 FT-IR spectrometer from Japan, at the College of Science at Qassim University.

#### 4.1.2. Experimental

Compounds 2–5 were synthesized according to the reported methods (see SI for the experimental and analytical details).^35–37^ See also SI for copies of IR, ^1^H-NMR, and ^13^C-NMR for all the synthesized compounds (6a–j).

#### 4.1.3. General procedure for the synthesis of diselenide-linked imidazolone (6a–j)

To a solution of the respective oxazolone (0.02 mol) in glacial acetic acid (15 mL), 4,4′-diselenediyldianiline (3) (0.02 mol) and freshly fused sodium acetate (0.025 mol) were added. The reaction mixture was refluxed for 6–18 h and monitored by TLC until complete consumption of the oxazolone was observed. After completion, the mixture was allowed to cool to room temperature, and the solid formed was filtered off. The obtained precipitate was then recrystallized from an appropriate solvent to afford the corresponding imidazolones 6a–j as pure crystalline products.

##### 4.1.3.1. 3,3′-(Diselanediylbis(4,1-phenylene))bis(5-benzylidene-2-phenyl-3,5-dihydro-4*H*-imidazol-4-one)one (6a)

Bage powder; yield: 88%; m.p. 98–99 °C (EtOH). FT-IR (KBr, cm^−1^): 1715 (C=O stretching, cyclic amide), 1639 (C=N stretching); ^1^H-NMR (DMSO-d_6_, 400 MHz), d (ppm): 8.16 (S, S, 4H, 2H(CH=C), 2H(Ar–H)), 8.16 (d, 2H, Ar–H), 7.6 (t, 2H, Ar–H), 7.5 (t, 2H, Ar–H), 7.4 (m, 12H, Ar–H), 7.3 (t, 4H, Ar–H), 7.1 (m, 4H, Ar–H); ^13^C-NMR (DMSO-d_6_, 100 MHz) d: 167.4, 163.56, 161.1, 138.8, 136.1, 134.6, 134.2, 133.6, 132.8, 132.1, 131.2, 129.8, 129.5, 129, 128.2, 125.6, 120.6.

##### 4.1.3.2. 3,3′-(Diselanediylbis(4,1-phenylene))bis(5-(4-chlorobenzylidene)-2-phenyl-3,5-dihydro-4*H*-imidazol-4-one) (6b)

Yellow crystals; yield: 60%; m.p. 230–232 °C (EtOH). FT-IR (KBr, cm^−1^): 2980 (CH–Ar), 1711 (C=O stretching, cyclic amide), 1641 (C=N stretching); ^1^H-NMR (DMSO-d_6_, 400 MHz), d (ppm): 8.40 (S, 4H, 2H(CH=C), 2H(Ar–H)), 7.6 (d, 2H, Ar–H), 7.5 (d, 4H, Ar–H), 7.4 (t, 8H, Ar–H), 7.3 (t, 4H, Ar–H), 7.2 (m, 6H, Ar–H); ^13^C-NMR (DMSO-d_6_, 100 MHz) d: 161.5, 139.2, 136.1, 135.6, 134.3, 133.4, 132.2, 129.4, 128, 128.8, 126.6.

##### 4.1.3.3. 3,3′-(Diselanediylbis(4,1-phenylene))bis(5-(4-nitrobenzylidene)-2-phenyl-3,5-dihydro-4*H*-imidazol-4-one) (6c)

Yellow crystals; yield: 93%; m.p. 250–252 °C (AcOH). FT-IR (KBr, cm^−1^): 1711.12 (C=O stretching, cyclic amide), 1640 (C=N stretching); ^1^H-NMR (DMSO-d_6_, 400 MHz), d (ppm): 8.24 (S, 4H, 2H(CH=C), 2H(Ar–H), 8.00 (d, 4H, Ar–H), 7.8 (d, 4H, Ar–H), 7.7 (d, 4H, Ar–H), 7.6 (t, 4H, Ar–H), 7.5 (t, t, 10H, Ar–H), 7.14 (s, 2H, Ar–H); ^13^C-NMR (DMSO-d_6_, 100 MHz) d: 166.5, 164.6, 146.9, 142, 140.01, 134.8, 133.6, 133.4, 132.5, 130.7, 128.9, 128.4, 128.09, 125.11, 124.68, 124.1, 121.5, 121.1.

##### 4.1.3.4. 3,3′-(Diselanediylbis(4,1-phenylene))bis(5-(2-nitrobenzylidene)-2-phenyl-3,5-dihydro-4*H*-imidazol-4-one) (6d)

Deep yellow crystals; yield: 85%; m.p. 124–125 °C (EtOH). FT-IR (KBr, cm^−1^): 1728.6 (C=O stretching, cyclic amide), 1634 (C=N stretching); ^1^H-NMR (DMSO-d_6_, 400 MHz), d (ppm): 8.15 (S, 2H, (CH=C), 8.13 (t, 2H, Ar–H), 7.9 (t, 2H, Ar–H), 7.5 (m, 12H, Ar–H), 7.4 (t, 4H, Ar–H), 7.2–7.35 (m, 6H, Ar–H); ^13^C-NMR (DMSO-d_6_, 100 MHz) d: 169.8, 163.2, 149.8, 141.09, 140.4, 136.2, 134.2, 133.8, 132.4, 132.16, 131.2, 130.9, 129.5, 129.2, 128.9, 128.3, 125.3, 121.8, 120.6, 120.2.

##### 4.1.3.5. 3,3′-(Diselanediylbis(4,1-phenylene))bis(2-phenyl-5-(3,4,5-trimethoxybenzylidene)-3,5-dihydro-4*H*-imidazol-4-one) (6e)

Orange crystals; yield: 83%; m.p. 220–222 °C (EtOH). FT-IR (KBr, cm^−1^): 1714.15 (C=O stretching, cyclic amide), 1634 (C=N stretching); ^1^H-NMR (DMSO-d_6_, 400 MHz), d (ppm): 3.7 (s, 6H, 2-OCH_3_), 3.8 (s, 12H, 4-OCH_3_), 7.27 (m, 4H, Ar–H), 7.4 (t, 4H, Ar–H), 7.5 (m, 8H, Ar–H),7.6 (d, 2H, Ar–H), 7.66 (d, 4H, Ar–H), 7.8 (s, 2H, CH=C); ^13^C-NMR (DMSO, 100 MHz) d: 56.15, 60.7, 110.5, 128.5, 129.1, 129.5, 130.01, 130.67, 132.0, 132.14, 133.8, 134.6, 134.7, 137.9, 140.4, 153.2, 160.2, 169.9.

##### 4.1.3.6. 3,3′-(Diselanediylbis(4,1-phenylene))bis(5-(benzo[*d*][1,3]dioxol-5-ylmethylene)-2-phenyl-3,5-dihydro-4*H*-imidazol-4-one) (6f)

Yellow crystals; yield: 95%; m.p. 170–172 °C (AcOH). FT-IR (KBr, cm^−1^): 1716.4 (C=O stretching, cyclic amide), 1640 (C=N stretching); ^1^H-NMR (DMSO-d_6_, 400 MHz), d (ppm): 7.2 (s, 4H, 2-OCH̲_2_O–), 8.13 (t, 2H, Ar–H), 8.3 (m, 6H, Ar–H), 8.6 (m, 6H, Ar–H),8.7 (t, 3H, Ar–H), 9 (m, 4H, Ar–H), 9.1 (s, 2H, CH=C); ^13^C-NMR (DMSO-d_6_, 100 MHz) d: 99.06, 105.3, 106, 116.2, 120, 123.4, 123.9, 124.3, 125.2, 125.4, 126.2, 127.8, 128.9, 132.4.

##### 4.1.3.7. 3,3′-(Diselanediylbis(4,1-phenylene))bis(5-(furan-2-ylmethylene)-2-phenyl-3,5-dihydro-4*H*-imidazol-4-one) (6g)

Yellow powder; yield: 75%; m.p. 118–119 °C (AcOH). FT-IR (KBr, cm^−1^): 1714.7(C=O stretching, cyclic amide), 1637.31 (C=N stretching); ^1^H-NMR (DMSO-d_6_, 400 MHz), d (ppm): 7.15 (t, 2H, Ar–H), 7.2 (m, 4H, Ar–H), 7.3 (m, 4H, Ar–H), 7.5 (m, 10H, Ar–H), 7.6 (t, 4H, Ar–H), 7.7 (d, 2H, Ar–H), 8.1 (s, 2H, CH=C); ^13^C-NMR (DMSO-d_6_, 100 MHz) d: 112.9, 114.5, 120.02, 128.14, 128.9, 129.1, 129.5, 130.6, 131.1, 132.1, 133.5, 133.8, 134.5, 136, 147.7, 147.7, 150.9, 169.4.

##### 4.1.3.8. 3,3′-(Diselanediylbis(4,1-phenylene))bis(2-phenyl-5-(thiophen-2-ylmethylene)-3,5-dihydro-4*H*-imidazol-4-one) (6h)

Deep yellow crystals; yield: 92%; m.p. 310–312 °C (AcOH). FT-IR (KBr, cm^−1^): 1730.8 (C=O stretching, cyclic amide), 1632.3 (C=N stretching); ^1^H-NMR (DMSO-d_6_, 400 MHz), d (ppm): 7.2 (t, 4H, Ar–H), 8.13 (t, 2H, Ar–H), 8.6 (d, 4H, Ar–H),8.6 (m, 8H, Ar–H), 8.9 (m, 4H, Ar–H), 9 (d, 2H, Ar–H), 9.4 (s, 2H, CH=C); ^13^C-NMR (DMSO-d_6_, 100 MHz) d: 73.9, 101.7, 102.9, 109.3, 112.3, 112.6, 114.8, 115.6, 119.6, 129.1, 129.9, 145.8, 152.7, 166.6, 168.2, 175.7.

##### 4.1.3.9. 3,3′-(Diselanediylbis(4,1-phenylene))bis(5-((10*H*-phenothiazin-2-yl)methylene)-2-phenyl-3,5-dihydro-4*H*-imidazol-4-one) (6i)

Deep blue crystals; yield: 65%; m.p. 198–200 °C (DMF). FT-IR (KBr, cm^−1^): 3330.2 (NH), 1732.8 (C=O stretching, cyclic amide), 1651.7 (C=N stretching); ^1^H-NMR (DMSO-d_6_, 400 MHz), d (ppm): 6.7 (m, 4H, Ar–H), 6.9 (t, 6H, Ar–H), 6.9 (t, 4H, Ar–H), 7.00 (t, 4H, Ar–H), 7.1 (s, 2H, Ar–H), 7.3 (s, 2H, Ar–H),7.5 (d, 4H, Ar–H), 7.6 (t, 6H, Ar–H), 7.9 (d, 2H, Ar–H), 7.9 (s, 2H, CH=C), 8.1 (d, 4H, Ar–H), 9.6 (s, 2H, –NH); ^13^C-NMR (DMSO-d_6_, 100 MHz) d: 114.4, 114.9, 115.6, 116.1, 117.1, 123.3, 123.5, 126.7, 127.8, 128.1, 128.3, 129.8, 130.2, 140, 147.7, 190.4.

##### 4.1.3.10. 3,3′-((Diselanediylbis(4,1-phenylene))bis(5-oxo-2-phenyl-1,5-dihydro-4*H*-imidazole-1-yl-4-ylidene))bis(1-acetylindolin-2-one) (6j)

Brown crystals; yield: 60%; m.p. 124–125 °C (EtOH). FT-IR (KBr, cm^−1^): 1717.5 (C=O stretching, cyclic amide), 1694 
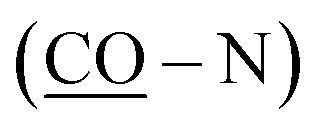
, 1637.4 (C=N stretching); ^1^H-NMR (DMSO-d_6_, 400 MHz), d (ppm): 2.09 (s, 6H, COCH̲_3_), 6.9 (t, 2H, Ar–H), 7.2 (d, 2H, Ar–H), 7.3 (d, 4H, Ar–H), 7.00 (t, 4H, Ar–H), 7.5 (m, 8H, Ar–H), 7.6 (m, 6H, Ar–H),7.66 (d, 2H, Ar–H), 7.9 (s, 2H, CH=C); ^13^C-NMR (DMSO-d_6_, 100 MHz) d: 224.5, 120.1, 120.4, 129.8, 133.6, 133.8, 134.5, 139.4, 140.1, 169.0.

### 4.2. Biological assays

#### 4.2.1. Cellular growth inhibition percentage (GI%) against different cancerous cell lines

All cancer cell lines used in this study were obtained from Vacsera (Giza, Egypt). The cytotoxic potential of the synthesized diselenide-linked imidazolone derivatives (6a–j) was evaluated using the SRB colorimetric assay^53,54^ as detailed in the SI. The compounds were tested against a panel of six human cancer cell lines, including HCT_116_, MCF7, HuH7, PC3, A549, and MG63, all obtained from the American Type Culture Collection (ATCC).

#### 4.2.2. Evaluation of cytotoxic inhibitory concentration 50 (IC_50_) against PC3, MCF7, A549, and HCT_116_ cells

To gain a deeper understanding of the cytotoxic efficacy of the synthesized diselenide-linked imidazolone derivatives (6a–j), their IC_50_s were determined. This evaluation was conducted on the cancer cell lines that exhibited the highest growth inhibition rates during the preliminary screening phase (details provided in the SI). Accordingly, IC_50_ values were determined against PC3, MCF7, A549, and HCT_116_ cell lines using the SRB assay, following the previously described protocol.^53,55,56^

#### 4.2.3. Protein expression of apoptosis and cell cycle-related proteins in A549 cells treated with 6g

To further investigate the apoptotic potential of the most active diselenide-linked imidazolone derivative, compound 6g, protein expression analysis was conducted using the A549 cell line. This experiment aimed to assess the expression levels of key apoptosis-related proteins and elucidate the molecular mechanisms underlying the compound's cytotoxic effects (details provided in the SI).^57,58^ Comparative analysis between treated and untreated cells was performed to identify significant alterations in protein expression, thereby providing insight into the pro-apoptotic pathways activated by this selenium-containing derivative.

#### 4.2.4. Evaluation of cell cycle arrest for 6g in the A549

To evaluate the impact of compound 6g on cell cycle progression, A549 lung cancer cells^59,60^ (2 × 10^5^ cells per well) were seeded into 12-well plates and treated with various concentrations of the test compound for different incubation periods. Following treatment, cells were harvested and fixed overnight in ice-cold 70% ethanol prepared in phosphate-buffered saline (PBS). After fixation, the cells were washed and resuspended in PBS containing 40 µg per mL propidium iodide, 0.1 mg per mL RNase A (Sigma, USA), and 0.1% Triton X-100. The suspensions were then incubated at 37 °C for 30 minutes in the dark to allow complete staining and RNA degradation. Subsequently, samples were analyzed using a flow cytometer (Becton Dickinson, San Jose, CA, USA) equipped with a 488 nm argon laser. The obtained data were used to quantify the distribution of cells within the G_0_/G_1_, S, and G_2_/M phases, providing insight into the cell cycle arrest induced by 6g.

### 4.3. Molecular docking

To further investigate the apoptotic potential of compound 6g, a molecular docking study was conducted against the CDK6 target receptor using the AutoDock Vina^61^ and PyMOL.^62^ Analogue 6g was first prepared by energy minimization and optimization of partial charges.^63,64^ The PDB was searched, and the CDK6 receptor with ID (5L2I) was selected and prepared through hydrogenation (3D), correction, and energy minimization.^65^ The co-crystal inhibitor (palbociclib) of 5L2I was inserted as a positive reference to analogue 6g. Finally, a redocking procedure for the CDK6 co-crystal palbociclib clarified the validity of the applied software (RMSD < 2 Å).^66^

## Author contributions

Conceptualization: Ahmed A. Al-Karmalawy and Saad Shaaban; supervision: Marwa Abdel-Motaal, Saad Shaaban, and Ahmed A. Al-Karmalawy; data curation, visualization, methodology, and writing – review & editing: Marwa Abdel-Motaal, Saad Shaaban, Samia S. Hawas, Asma M. Elsharif, Marwa Sharaky, Fatema S. Alatawi, Mohamed E. Eissa, Arwa Omar Al Khatib, Hany M. Abd El-Lateef, Medhat Asem, and Ahmed A. Al-Karmalawy. Finally, all authors revised and approved the final submitted version of the manuscript.

## Conflicts of interest

The authors declared no conflict of interest.

## Funding

This work was supported by the Deanship of Scientific Research, Vice Presidency for Graduate Studies and Scientific Research, King Faisal University, Saudi Arabia [Grant No. KFU260238].

## Supplementary Material

RA-016-D5RA10063A-s001

## Data Availability

The data supporting this article have been included in the manuscript and the supplementary information (SI). Supplementary information is available. See DOI: https://doi.org/10.1039/d5ra10063a.
