# Supplementary material for: Targeting cell cycle and apoptotic pathways with newly synthesized diselenide-linked imidazolone analogues with strong CDK6-targeting potential
Source: RSC Adv. 2026 Feb 4;16(8):7066–79. doi: 10.1039/d5ra10063a (PMC12871525; doi:10.1039/d5ra10063a)
Supplement: RA-016-D5RA10063A-s001 [file RA-016-D5RA10063A-s001.pdf]

Supplementary Information (SI) for RSC Advances.
This journal is © The Royal Society of Chemistry 2026

Supplementary Information

Targeting Cell Cycle and Apoptotic Pathways with Newly Synthesized Diselenide-linked Imidazolone Analogues with Strong CDK6-targeting Potential

Marwa Abdel-Motaal[1,2,¥], Saad Shaaban[3,¥,*], Samia S. Hawas[4,¥], Asma M. Elsharif[5], Marwa Sharaky[6], Fatema S. Alatawi[7], Mohamed E. Eissa[8], Arwa Omar Al Khatib[9], Hany M. Abd El-Lateef[3], Medhat Asem[10], Ahmed A. Al-Karmalawy[11,4,*]

[1] Department of Chemistry, College of Science, Qassim University, Buraydah 51452, Qassim, Saudi Arabia. E
[2] Organic Chemistry Division, Department of Chemistry, College of Science, Mansoura University, Mansoura, Egypt.
[3] Department of Chemistry, College of Science, King Faisal University, Al-Ahsa 31982, Saudi Arabia.
[4] Department of Pharmaceutical Chemistry, Faculty of Pharmacy, Horus University-Egypt, New Damietta 34518, Egypt.
[5] Department of Chemistry, College of Science, Imam Abdulrahman Bin Faisal University, Dammam 31441, Saudi Arabia.
[6] Cancer Biology Department, Pharmacology Unit, National Cancer Institute (NCI), Cairo University, Cairo, Egypt.

[7] Department of Biochemistry, Faculty of Science, University of Tabuk, Tabuk, Saudi Arabia.
[8] Department of Chemistry, College of Science, Imam Mohammad Ibn Saud Islamic University (IMSIU), Riyadh 11623, Saudi Arabia.
[9] Faculty of Pharmacy, Al-Ahliyya Amman University, Amman, Jordan.

[10] Department of Civil Engineering, College of Engineering and Information Technology, Onaizah Colleges, Qassim 56447, Saudi Arabia.
[11] Department of Pharmaceutical Chemistry, College of Pharmacy, The University of Mashreq, Baghdad 10023, Iraq.

*Corresponding authors:

Ahmed A. Al-Karmalawy; Email: akarmalawy@horus.edu.eg

Saad Shaaban; Email: sibrahim@kfu.edu.sa

[¥] These authors contributed equally.

Chemistry

1. Materials and methods

General

The reagents (such as hippuric acid, aromatic aldehydes, acetic anhydride, sodium acetate, aniline, and SeO_2), solvents, and thin-layer chromatography (TLC) aluminum plates coated with silica gel (60 F254, Merck) utilized in the synthesis of the targets were purchased from Sigma-Aldrich. TLC was employed to monitor the completion of the reaction and to confirm the purity of the products. Visualization of the spots at TLC was achieved under UV light at 254 nm and 365 nm. Measuring the melting point ºC *via* the Stuart SMP30 apparatus, and no corrections were applied. NMR spectra of the synthesized targets were assessed using a Bruker NMR spectrometer (at Faculty of Pharmacy, Mansoura University in Mansoura, Egypt) operating at frequencies of 400 MHz (^{1}H-NMR) and 100 MHz (^{13}C-NMR). TMS (tetramethylsilane) was used as reference material, and the synthesized compounds were dissolved in DMSO-d6 or in TFA (d-trifluoroacetic acid) in δ (ppm). Measurement of IR spectra *via* Shimadzu Model 8000 FT-IR spectrometer from Japan, at the College of Science at Qassim University.

Synthesis of OSe compounds 4-Selenocyanatoaniline (2) and 4,4′-Diselanediyldianiline (3) [1]

Compound **3** was synthesized over two steps: The first step includes the synthesis of 4-selenocyanatobenzenamine **2**. Briefly, SeO_2 (6 mmol) was added under stirring to a solution of malononitrile (3 mmol) in DMSO (15 mL). The mixture was stirred at room temperature for 15 min in order to obtain triselenium dicyanide. When the exothermic reaction had finished, aniline (5 mmol) was added. The mixture was stirred for 20 min. Water (150 mL) was added to the reaction mixture, and the resulting precipitate (4-selenocyanatobenzenamine) was filtered off, dried, and used without further purifications.

4-Selenocyanatoaniline (**2**) was obtained as a yellow solid (88 % yield). Mp: 73–74 °C. ^{1}H NMR (400 MHz, $CDCl_3$) δ 7.44 (d, J = 8.4 Hz, 2H, Ar-H), 6.64 (d, J = 8.4 Hz, 2H, Ar-H), 3.95 (s, 2H, NH_2).

In the second step, to a solution of 4-selenocyanatobenzenamine (1 mmol) in absolute ethanol (40 ml), NaOH (2 mmol) was added in small portions with caution. The mixture was stirred at room temperature for 2 h. Cold water (50 mL) was added, and the formed solid was filtered, washed with water (three times, 50 mL each), and dried under vacuum.

4,4′-Diselanediyldianiline (**3**) was obtained as pale-yellow crystals (82 % yield. Mp: 78–80 °C. ^{1}H NMR (400 MHz, $CDCl_3$) δ 7.3 (m, 4H, Ar-H), 6.5 (m, 4H, Ar-H), 3.7 ppm (s, 4H, NH_2).

Synthesis of oxazolones 5a-k

Oxazolones (5a-k) were synthesized in good yields (85-90%) according to previously described methods [2-4]. In brief, hippuric acid (0.05 mol) was dissolved in acetic anhydride (10 ml). To this solution, freshly fused sodium acetate (0.1 mol) and an appropriate benzaldehyde and its derivatives, such as *p*-chloro, *p*-nitro, 3,4,5-trimethoxy, and 2-nitrobenzaldehyde, besides heterocyclic aldehydes such as furfural, thiophene-2-aldehyde, phenothiazine-5-aldehyde, piperonal, and isatin, were added. The mixture was refluxed for 2-3 h in a water bath. After monitoring the reaction mixture by TLC (pet. Ether - ethyl acetate), which affirms the completion of the reaction, the reaction mixture was poured into ice-water with vigorous stirring, and the solid obtained was recrystallized from ethanol or acetic acid to give the pure oxazolones **5a-j.**

4-benzylidene-2-phenyloxazol-5(4H)-one (5a)

Faint yellow powder (EtOH); Yield: 83%; FT-IR (KBr, cm^{-1}): stretching1794 (-C=O lactone), 1654 (-C=N).

4-(4-chlorobenzylidene)-2-phenyloxazol-5(4H)-one (5b)

Faint yellow crystals (EtOH); Yield: 91%; Mp: 186–87 °C; FT-IR (KBr, cm^{-1}): stretching1791 (-C=O lactone), 1654 (-C=N).

4-(4-nitrobenzylidene)-2-phenyloxazol-5(4H)-one (5c)

Yellow crystals (AcOH); Yield: 95%; Mp: 183–85 °C; FT-IR (KBr, cm^{-1}): stretching1796 (-C=O lactone), 1654.9 (-C=N).

4-(2-nitrobenzylidene)-2-phenyloxazol-5(4H)-one (5d)

Yellow crystals (AcOH); Yield: 91%; Mp: 154–5 °C; FT-IR (KBr, cm^{-1}): stretching1791 (-C=O lactone), 1653 (-C=N).

2-phenyl-4-(3,4,5-trimethoxybenzylidene)oxazol-5(4H)-one (5e)

Shiny yellow needles (AcOH); Yield: 94%; Mp: 156–7 °C; FT-IR (KBr, cm^{-1}): stretching1780 (-C=O lactone), 1620 (-C=N).

4-(benzo[d][1,3]dioxol-5-ylmethylene)-2-phenyloxazol-5(4H)-one (5f)

Yellow crystals (AcOH); Yield: 88%; Mp: 190–1 °C.

4-(furan-2-ylmethylene)-2-phenyloxazol-5(4H)-one (5g)

Shiny yellow needles (AcOH); Yield: 82%; Mp: 156–7 °C; FT-IR (KBr, cm^{-1}): stretching 1790 (-C=O lactone), 1654 (-C=N).

2-phenyl-4-(thiophen-2-ylmethylene)oxazol-5(4H)-one (5h)

Yellow crystals (AcOH); Yield: 92%; Mp: 172–3 °C; FT-IR (KBr, cm^{-1}): stretching 1787 (-C=O lactone), 1636 (-C=N).

4-((10H-phenothiazin-2-yl)methylene)-2-phenyloxazol-5(4H)-one (5i):

Blue crystals (AcOH); Yield: 75%; Mp: 178-9 ºC.

4-(2-oxoindolin-3-ylidene)-2-phenyloxazol-5(4H)-one (5j):

Orange crystals (AcOH); Yield: 88%; Mp: 280–2°C; FT-IR (KBr, cm^{-1}): stretching 1692 (-C=O lactone), 1617 (C=O amide), 1601 (-C=N).

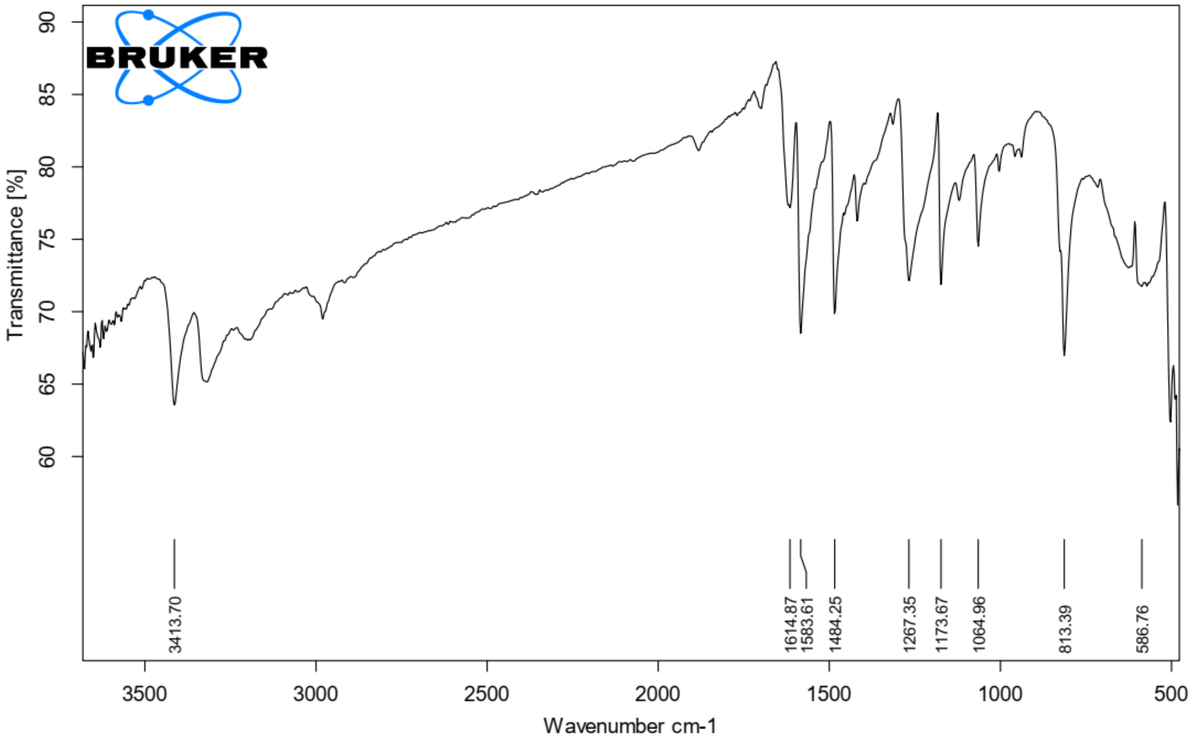

Figure S1. **FT- IR chart of compound 3**.

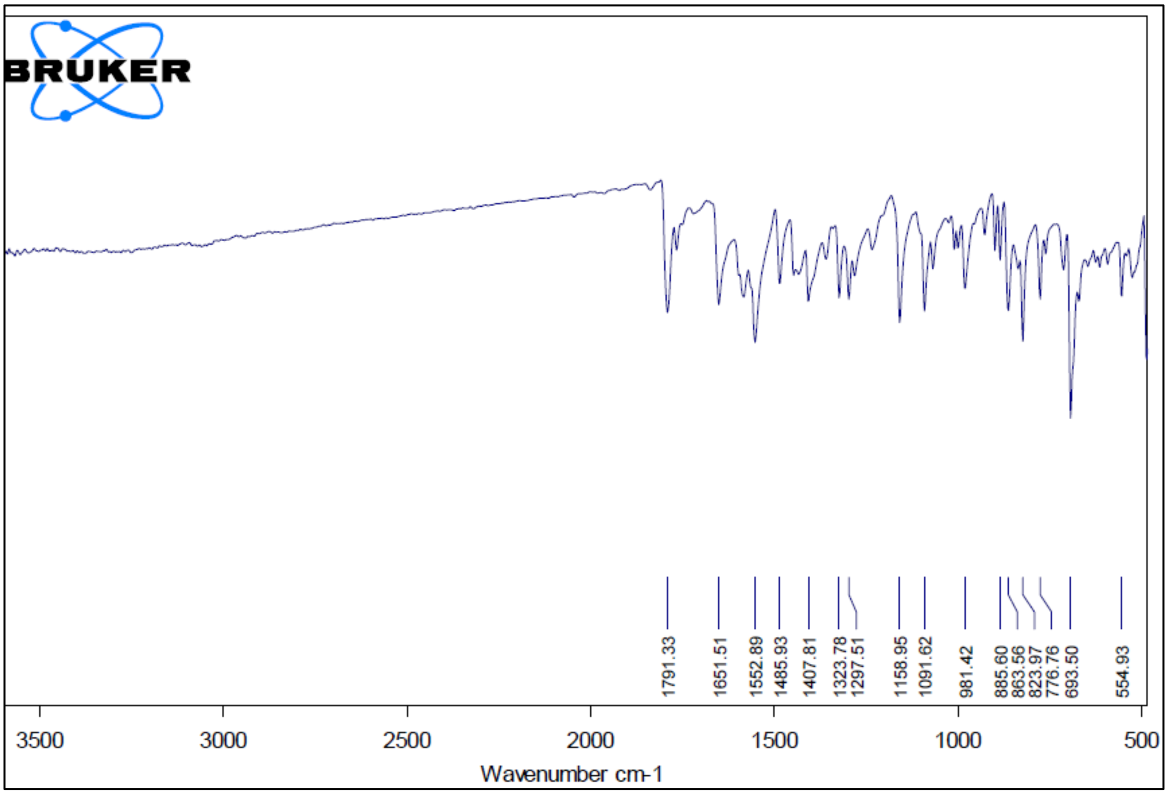

Figure S2. **FT- IR chart of compound 5b**.

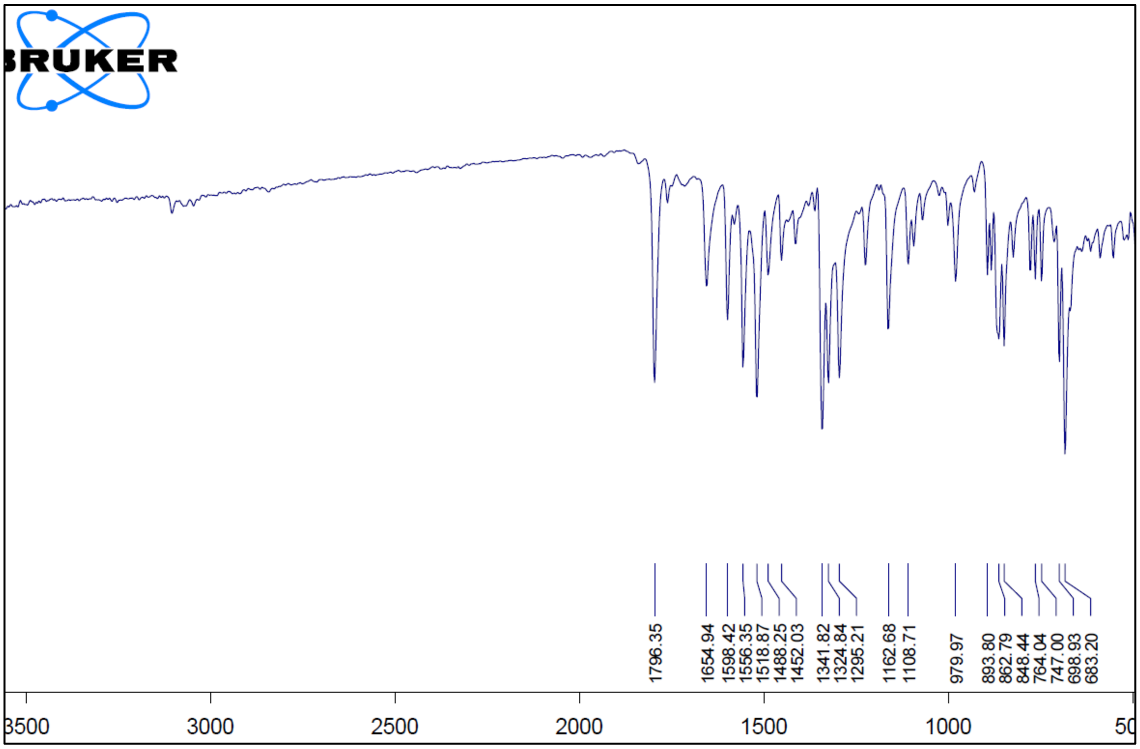

Figure S3. **FT- IR chart of compound 5c**.

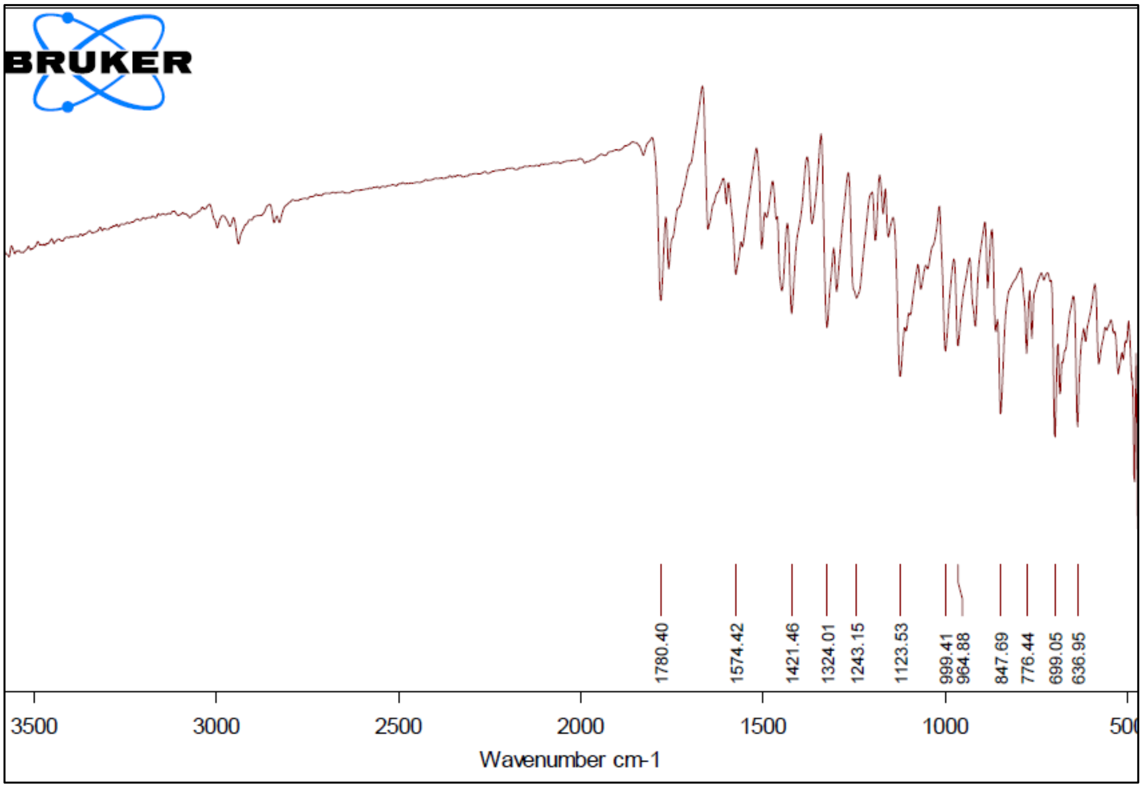

Figure S4. **FT- IR chart of compound 5e**.

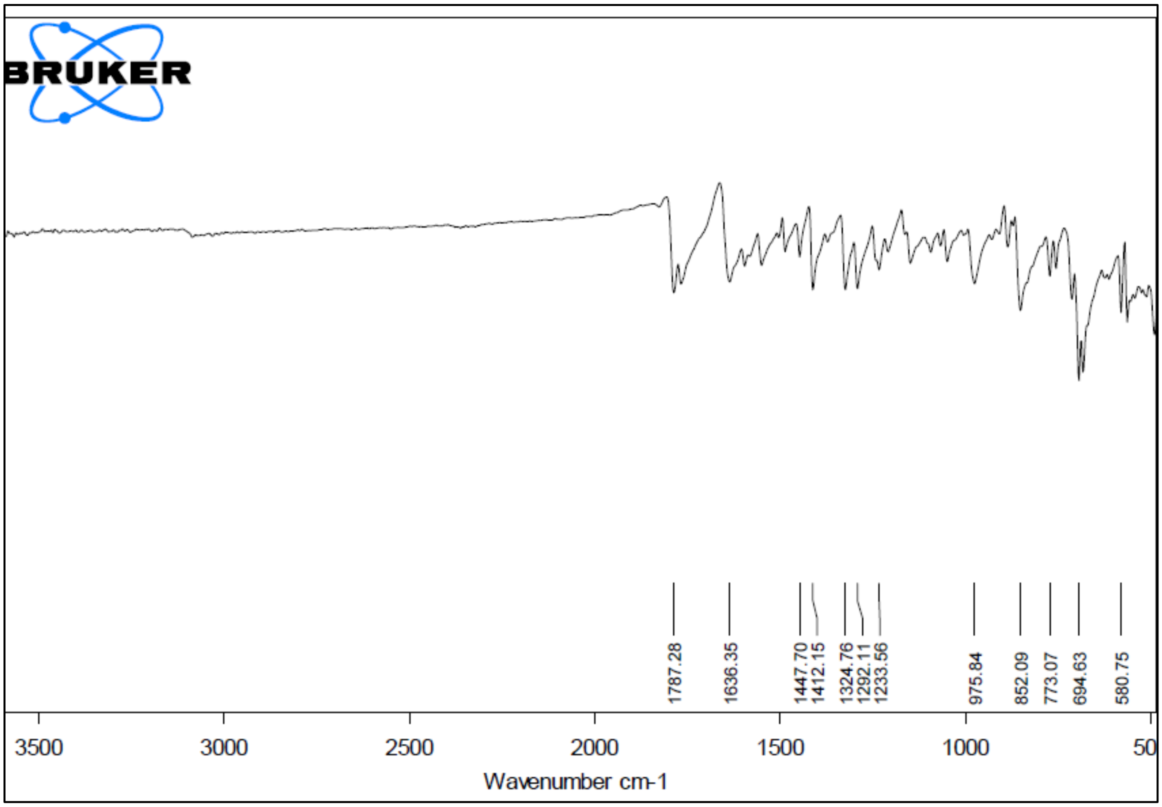

Figure S5. FT- IR chart of compound 5h.

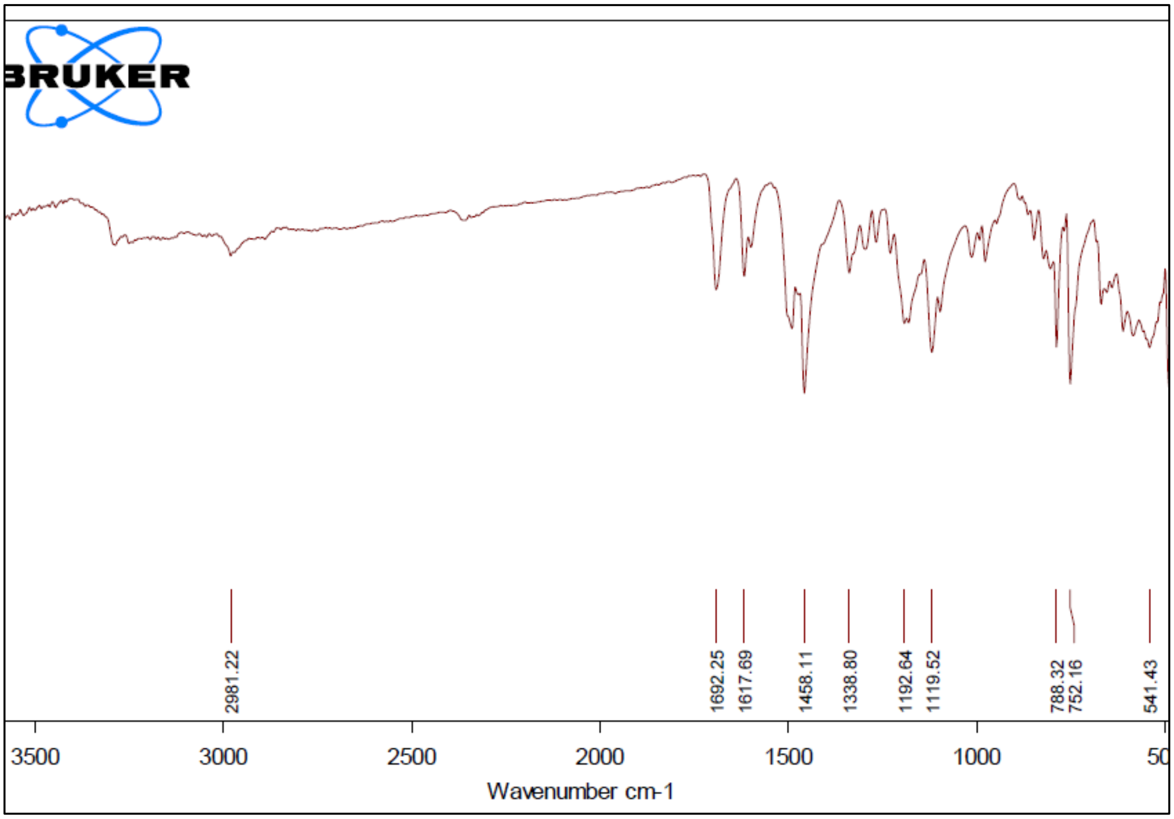

Figure S6. FT- IR chart of compound 5j.

3,3'-(diselanediylbis(4,1-phenylene))bis(5-benzylidene-2-phenyl-3,5-dihydro-4H-imidazol-4-one)one (6a)

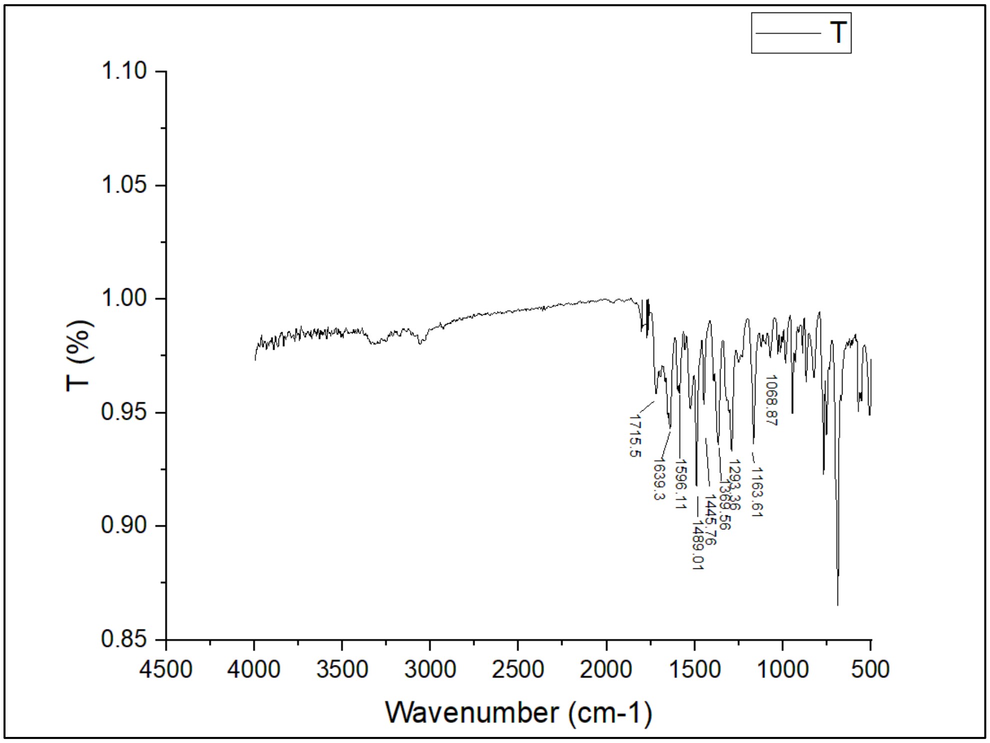

Figure S7. FT- IR chart of **6a**

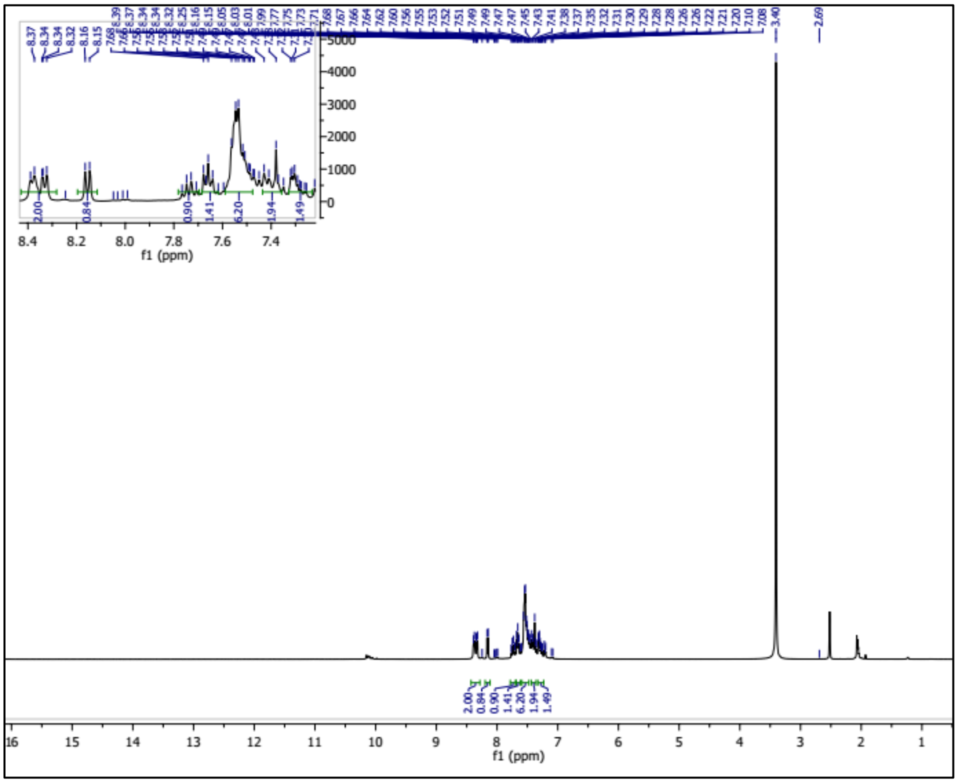

Figure S8. ^{1}HNMR chart of compound **6a**

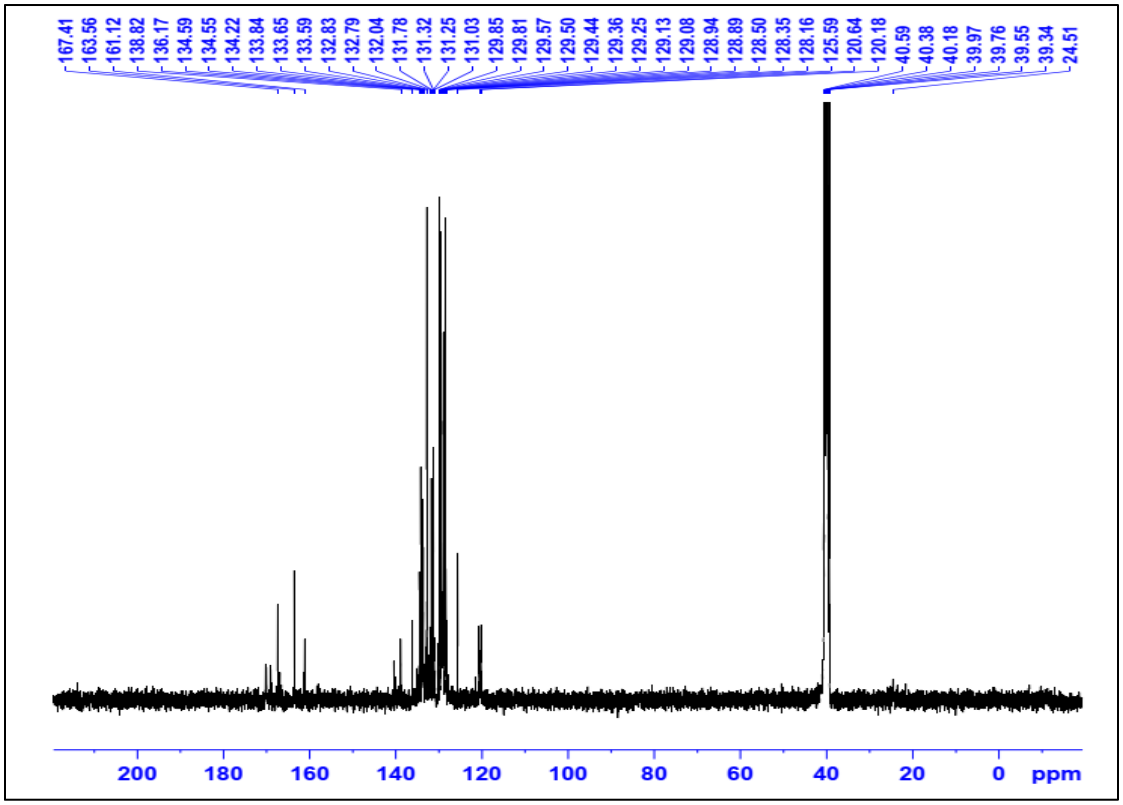

Figure S9. ^{13}CNMR chart of compound **6a**

3,3'-(diselanediylbis(4,1-phenylene))bis(5-(4-chlorobenzylidene)-2-phenyl-3,5-dihydro-4H-imidazol-4-one) (6b)

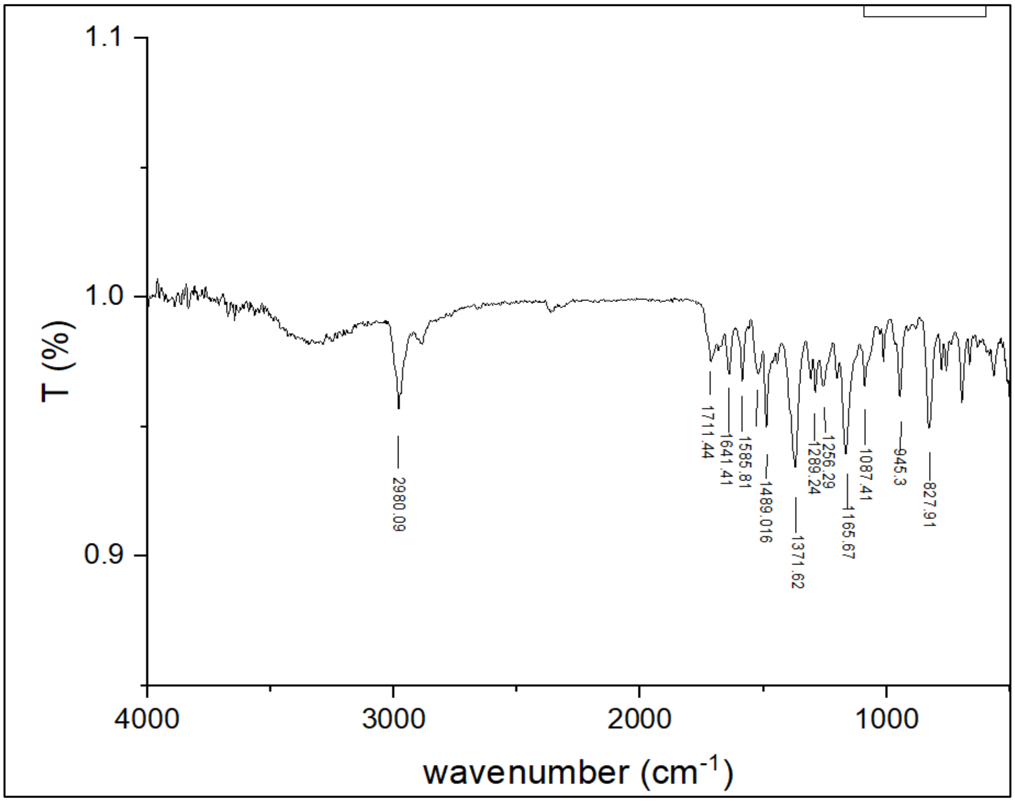

Figure S10. IR chart of compound **6b**

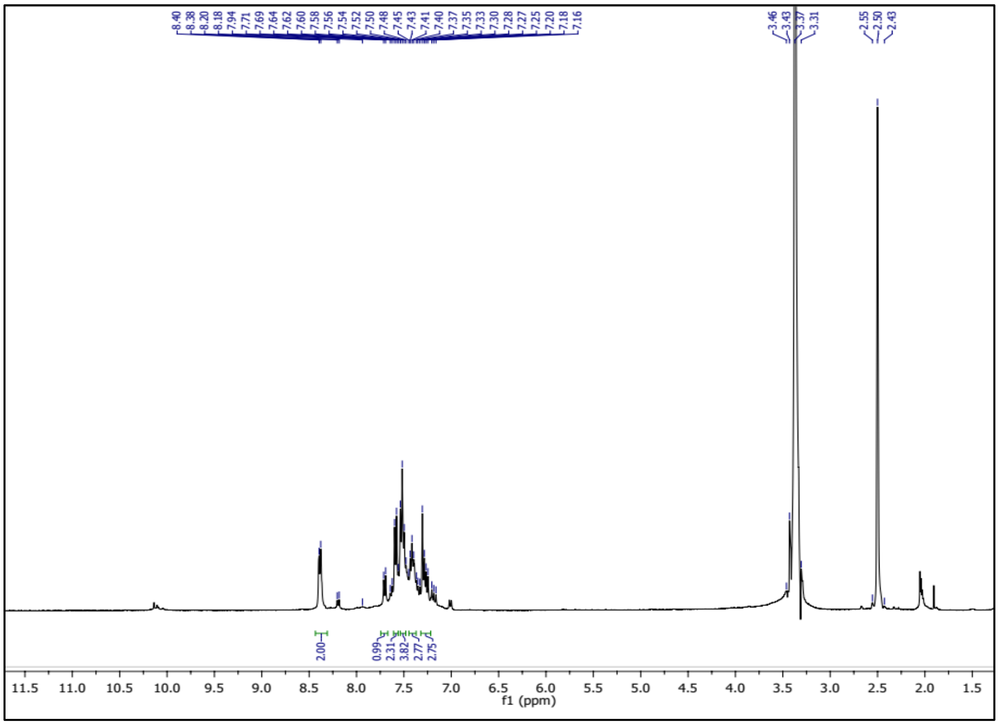

Figure S11. ^{1}HNMR chart of compound **6b**

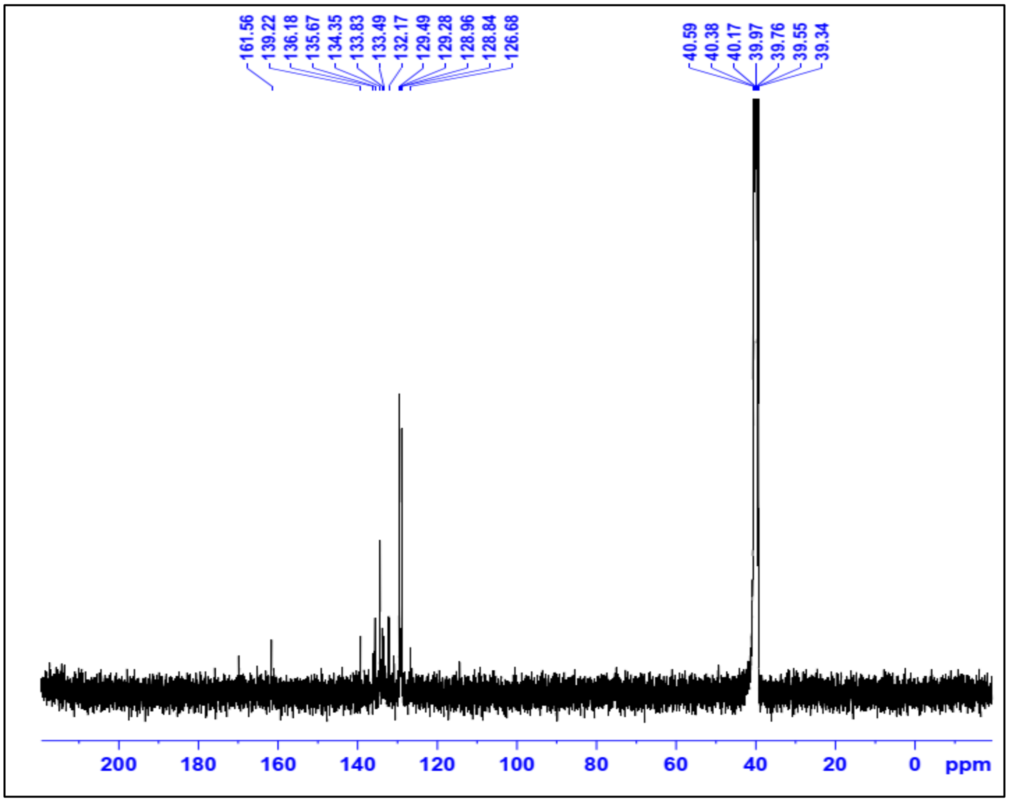

Figure S12. ^{13}CNMR chart of compound **6b**

3,3'-(diselanediylbis(4,1-phenylene))bis(5-(4-nitrobenzylidene)-2-phenyl-3,5-dihydro-4H-imidazol-4-one) (6c)

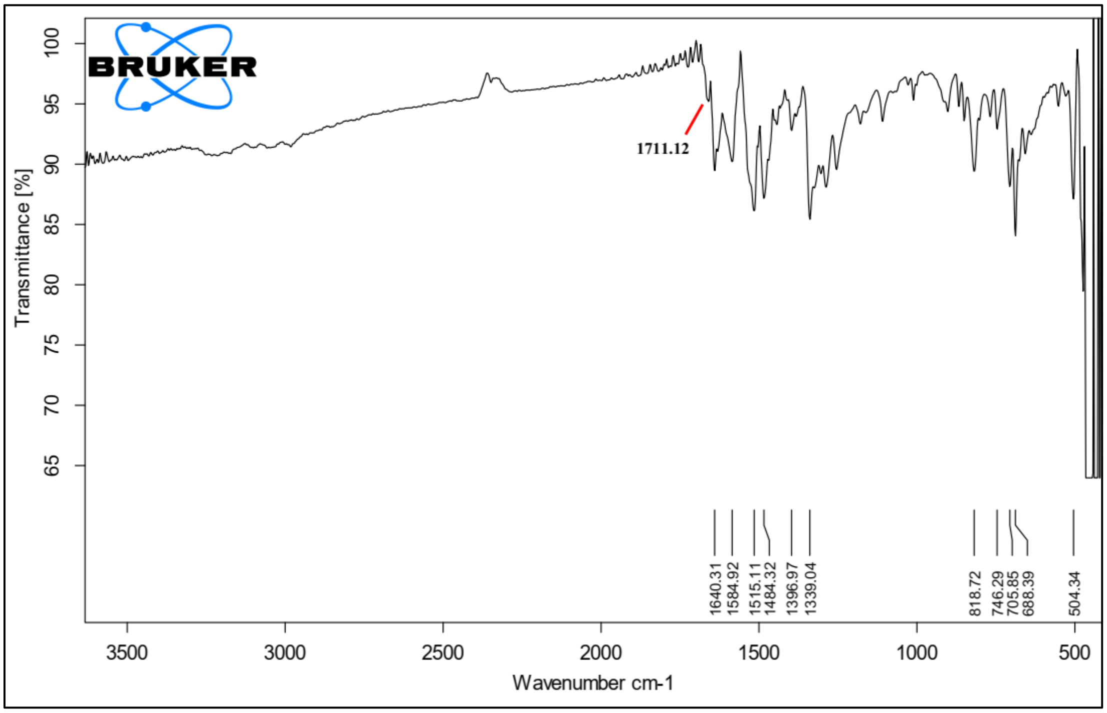

Figure S13. IR chart of compound **6c**

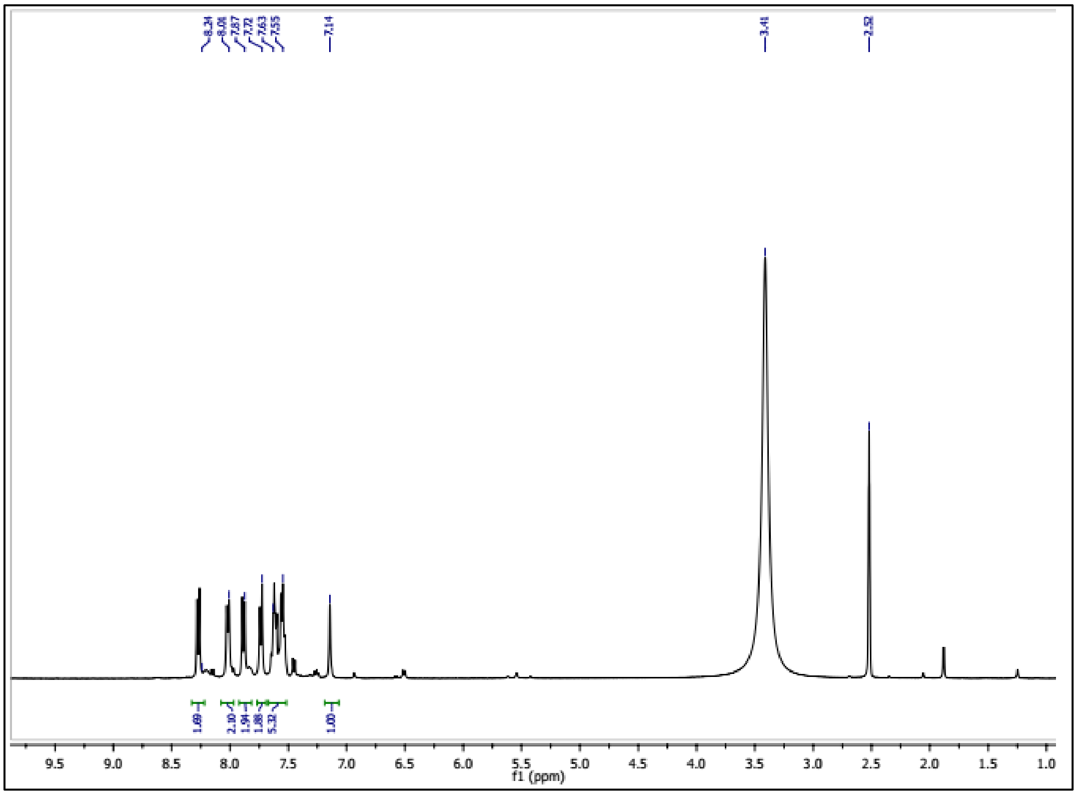

Figure S14. ^{1}HNMR chart of compound **6c**

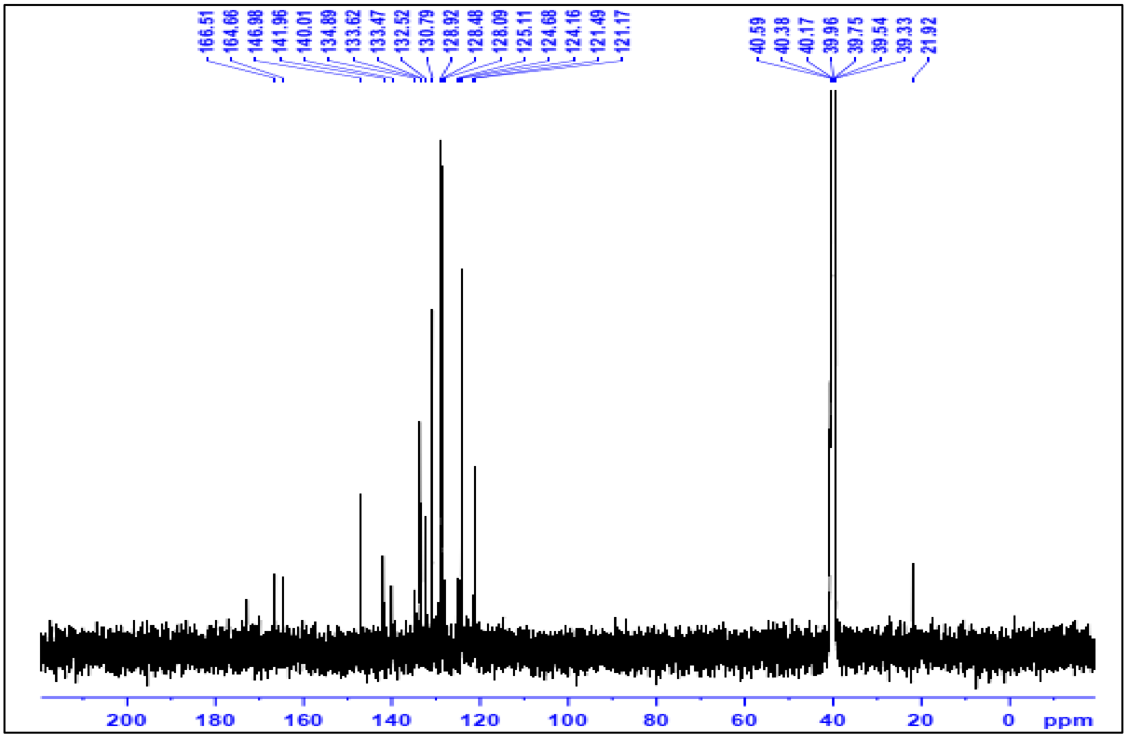

Figure S15. ^{13}CNMR chart of compound **6c**

3,3'-(diselanediylbis(4,1-phenylene))bis(5-(2-nitrobenzylidene)-2-phenyl-3,5-dihydro-4H-imidazol-4-one) (6d)

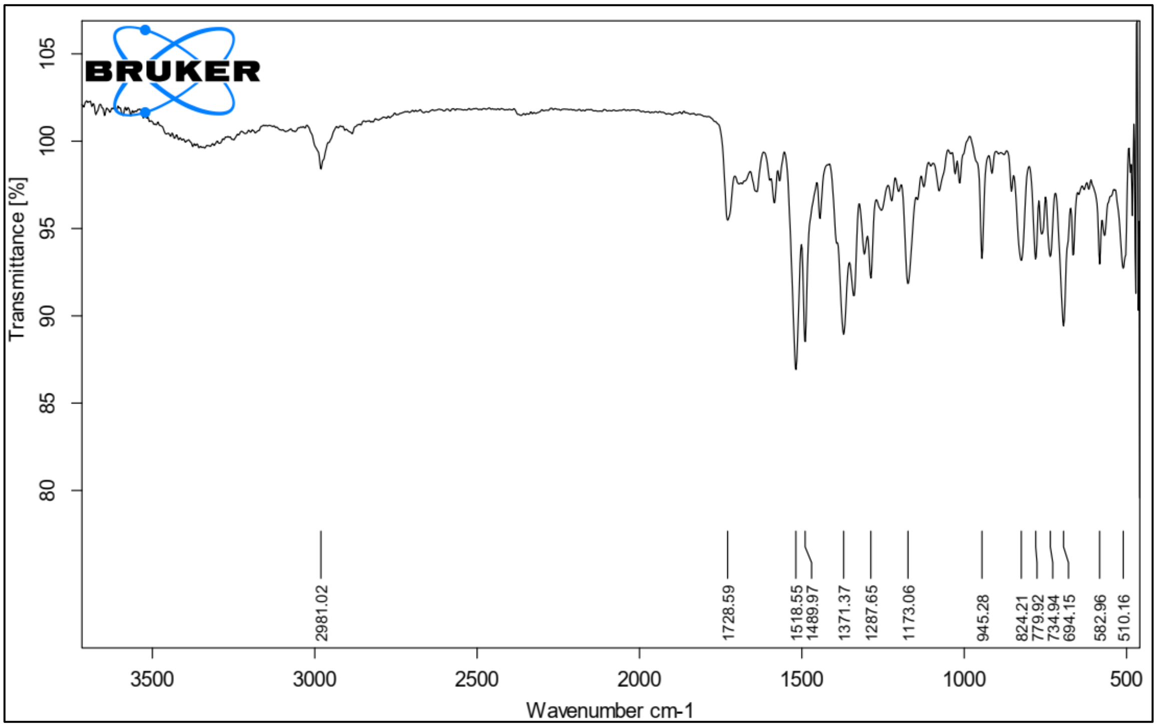

Figure S16. IR chart of compound **6d**

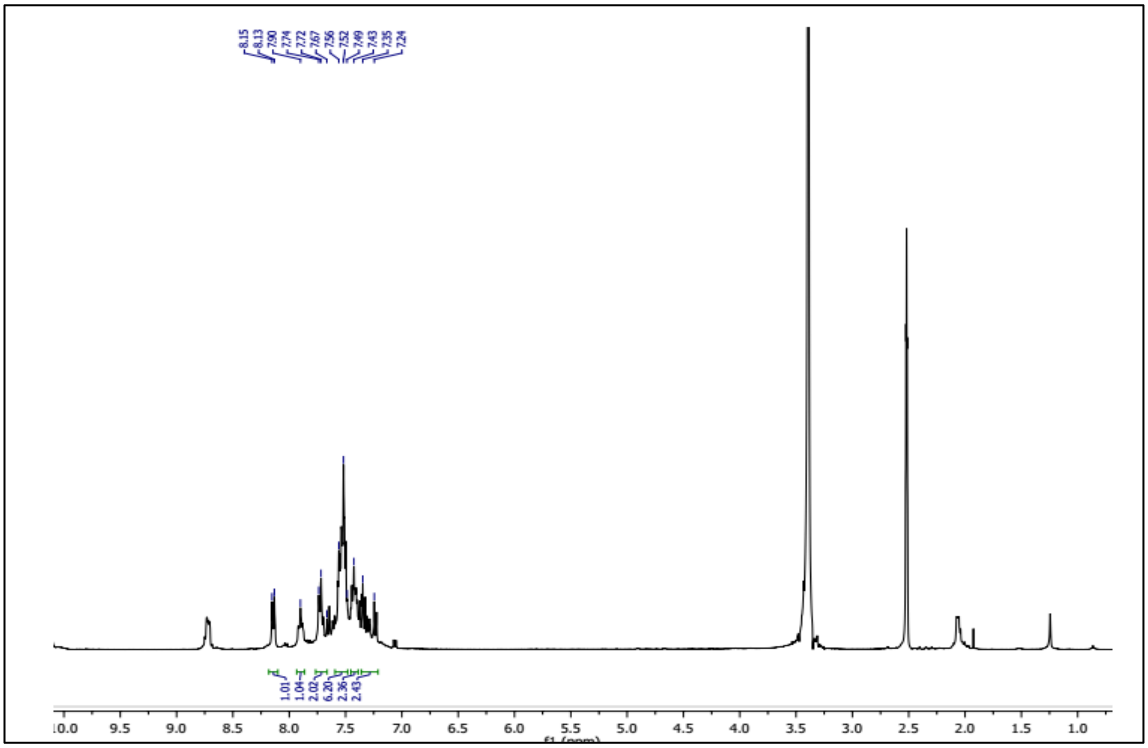

Figure S17. ^{1}HNMR chart of compound **6d**

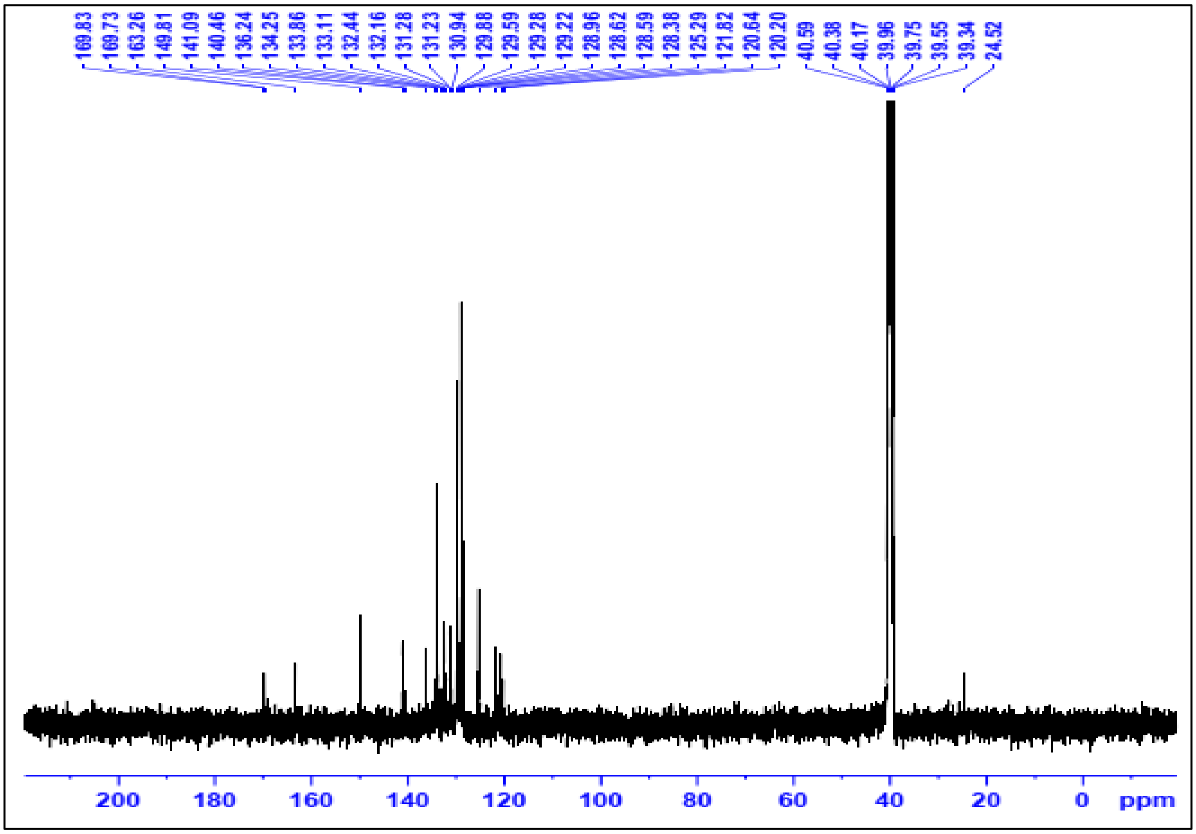

Figure S18. ^{13}CNMR chart of compound **6d**

3,3'-(diselanediylbis(4,1-phenylene))bis(2-phenyl-5-(3,4,5-trimethoxybenzylidene)-3,5-dihydro-4H-imidazol-4-one) (6e)

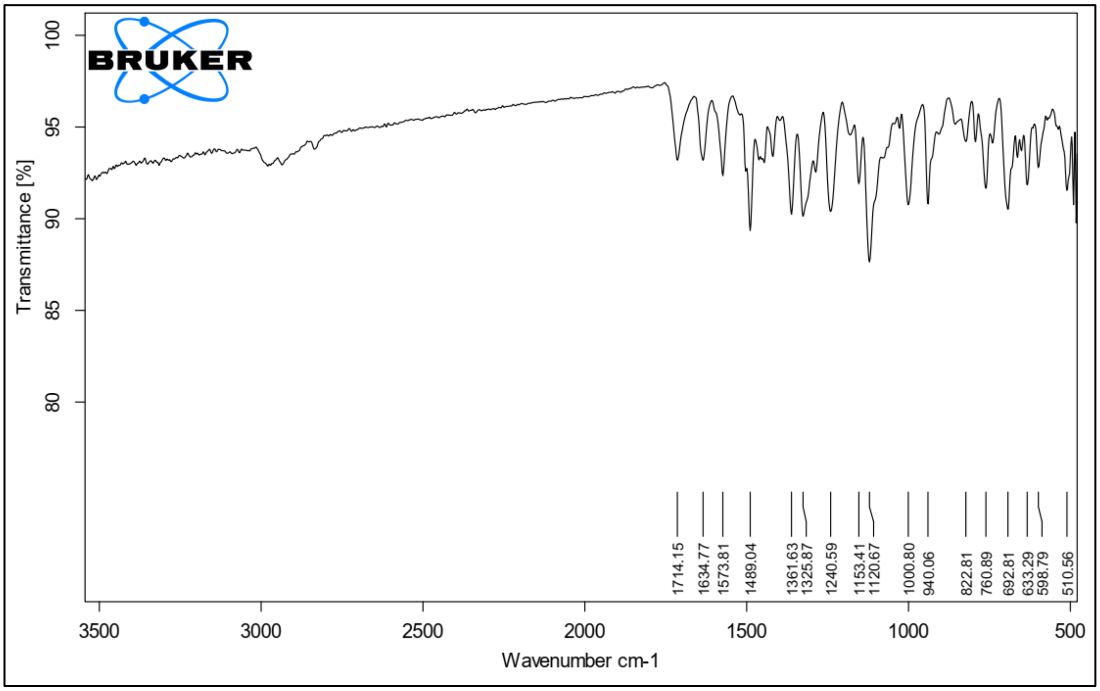

Figure S19. IR chart of compound **6e**

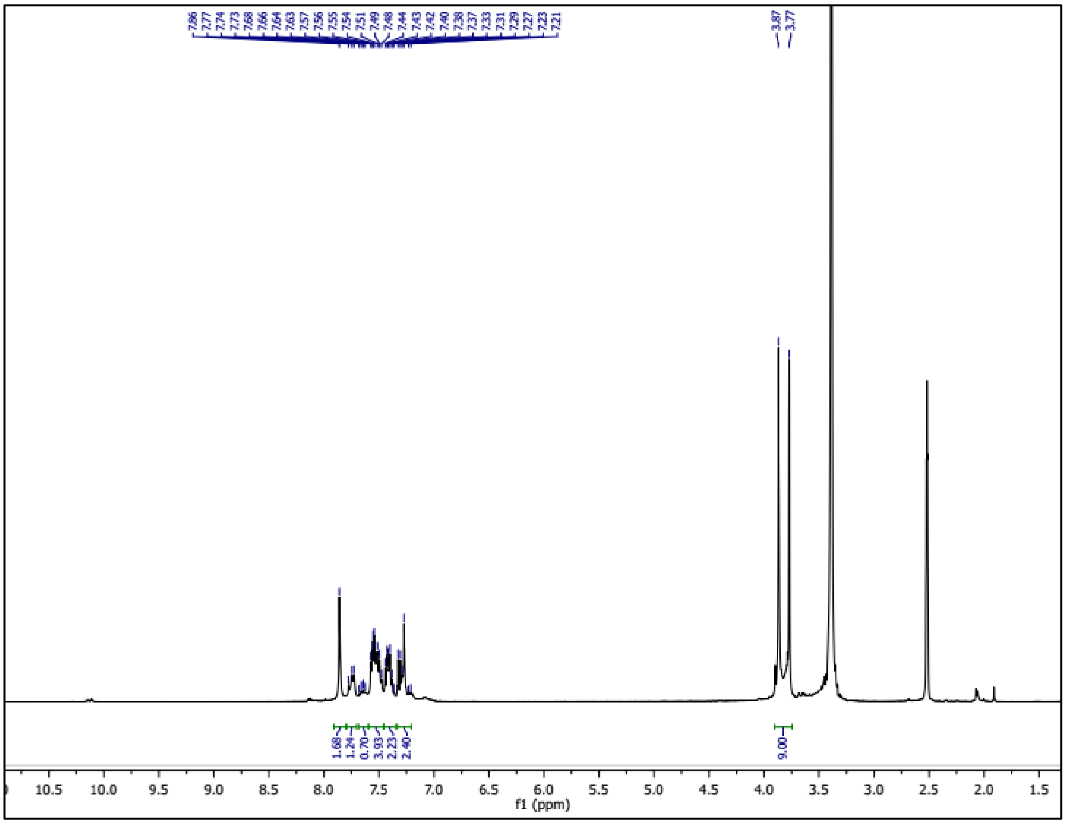

Figure S20. ^{1}HNMR chart of compound **6e**

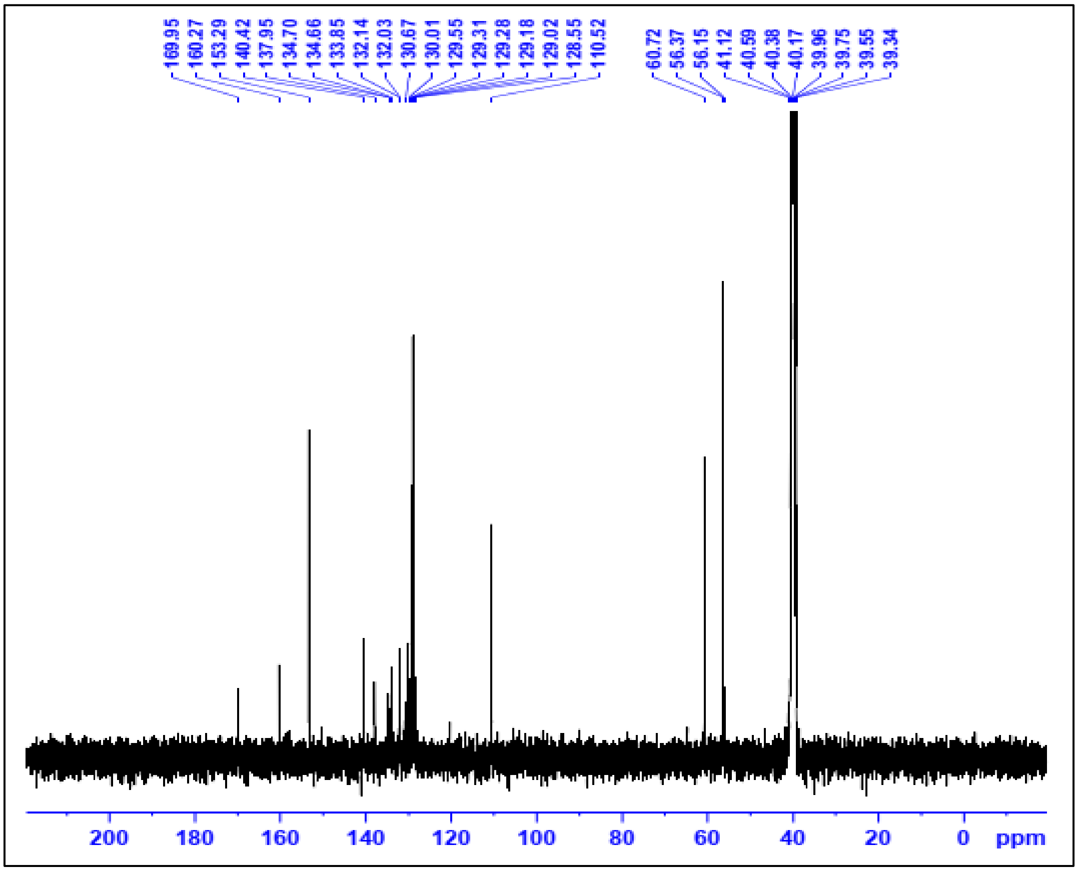

Figure S21. ^{13}CNMR chart of compound **6e**

3,3'-(diselanediylbis(4,1-phenylene))bis(5-(benzo[d][1,3]dioxol-5-ylmethylene)-2-phenyl-3,5-dihydro-4H-imidazol-4-one) (6f)

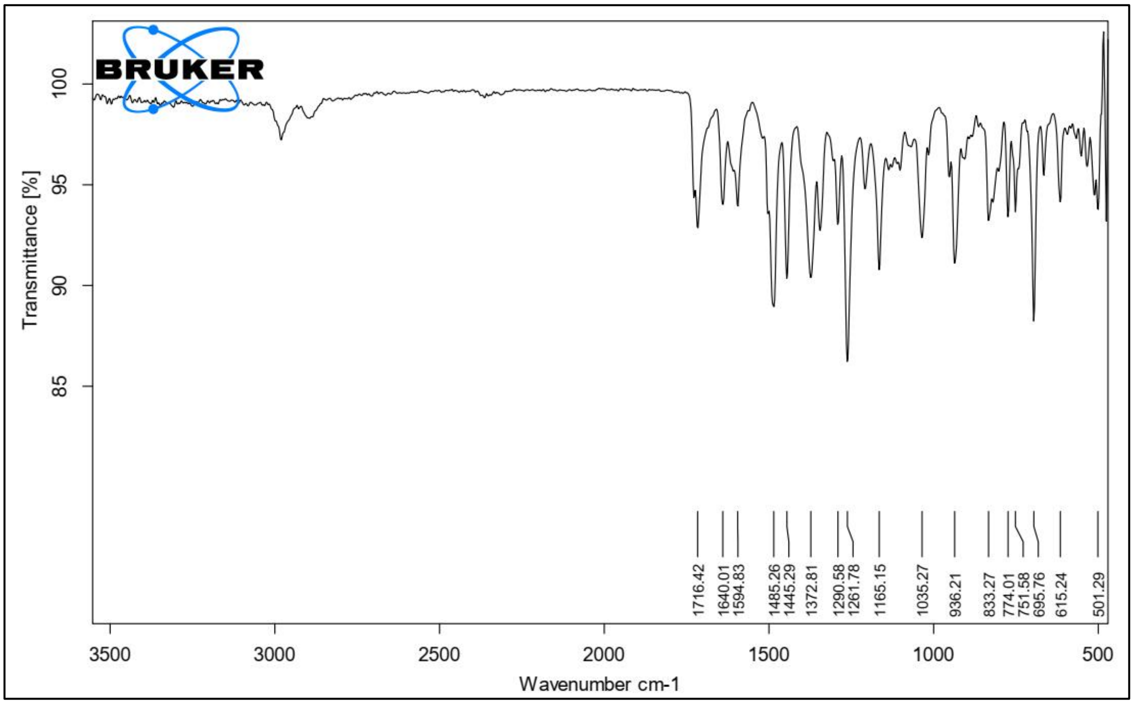

Figure S22. IR chart of compound **6f**

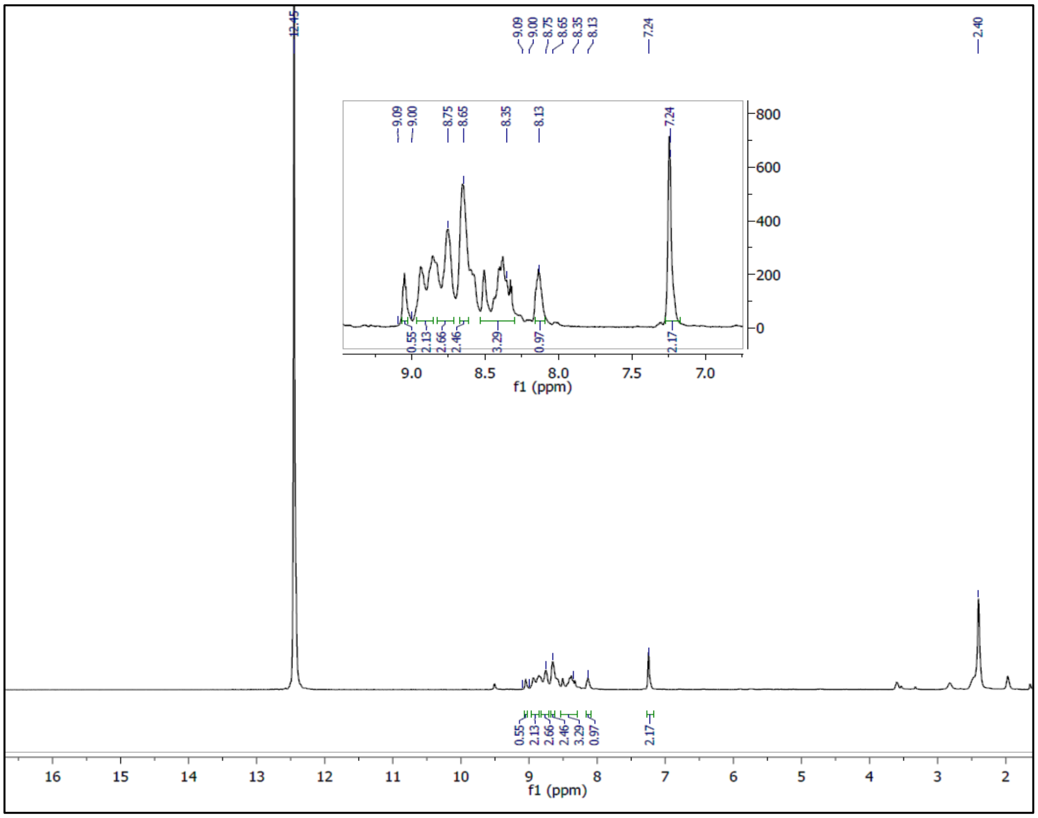

Figure S23. ^{1}HNMR chart of compound **6f**

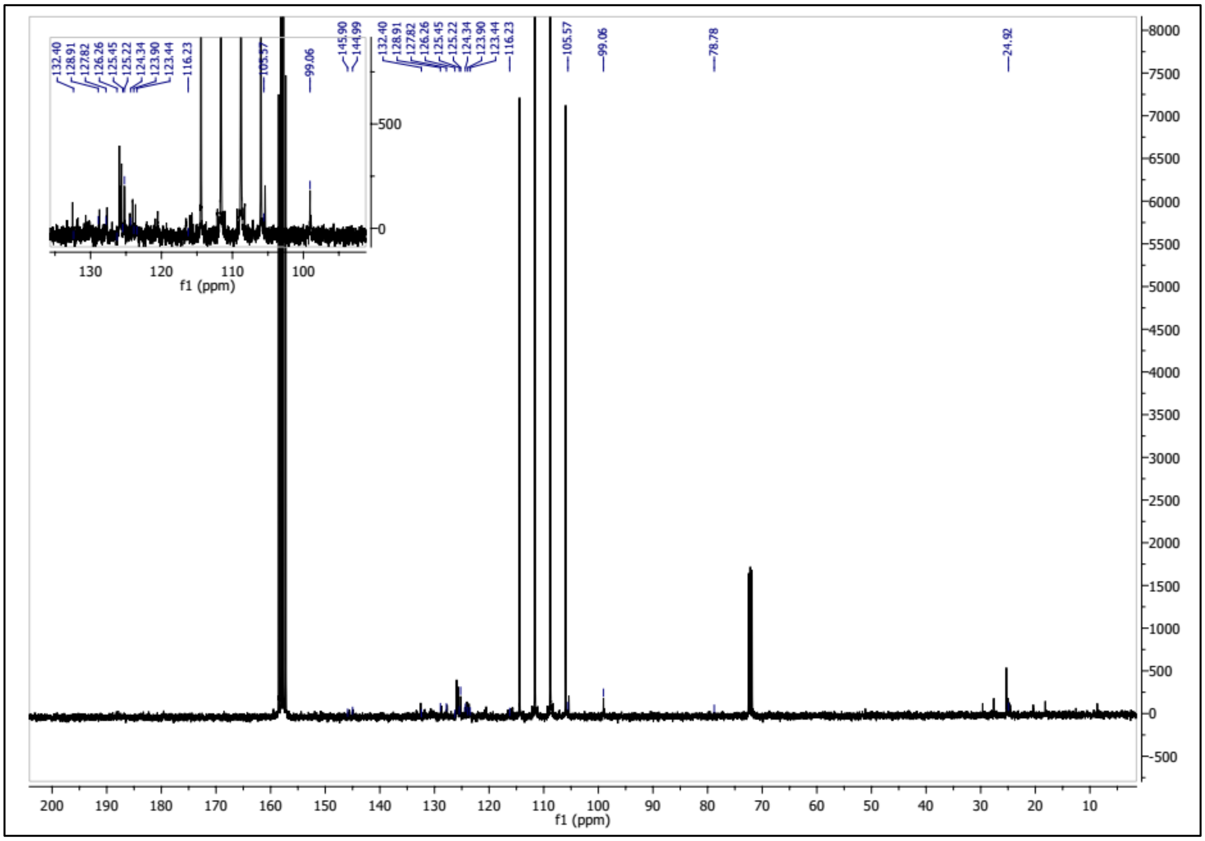

Figure S24. ^{13}CNMR chart of compound **6f**

3,3'-(diselanediylbis(4,1-phenylene))bis(5-(furan-2-ylmethylene)-2-phenyl-3,5-dihydro-4H-imidazol-4-one) (6g)

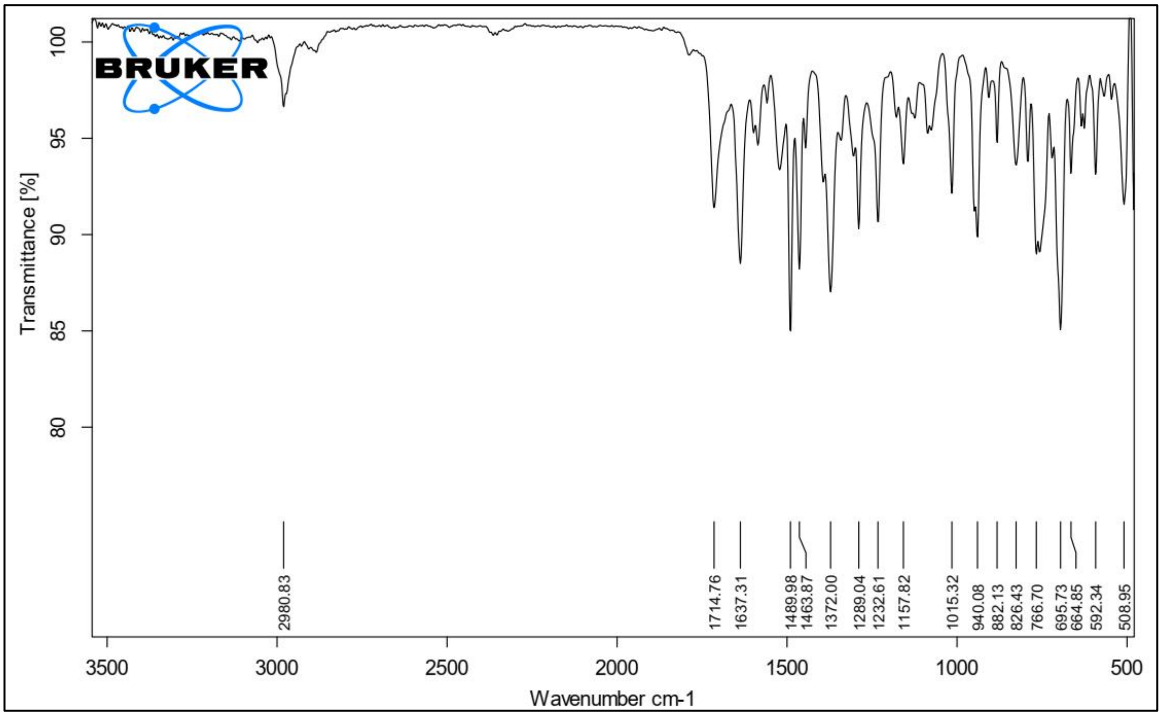

Figure S25. IR chart of compound **6g**

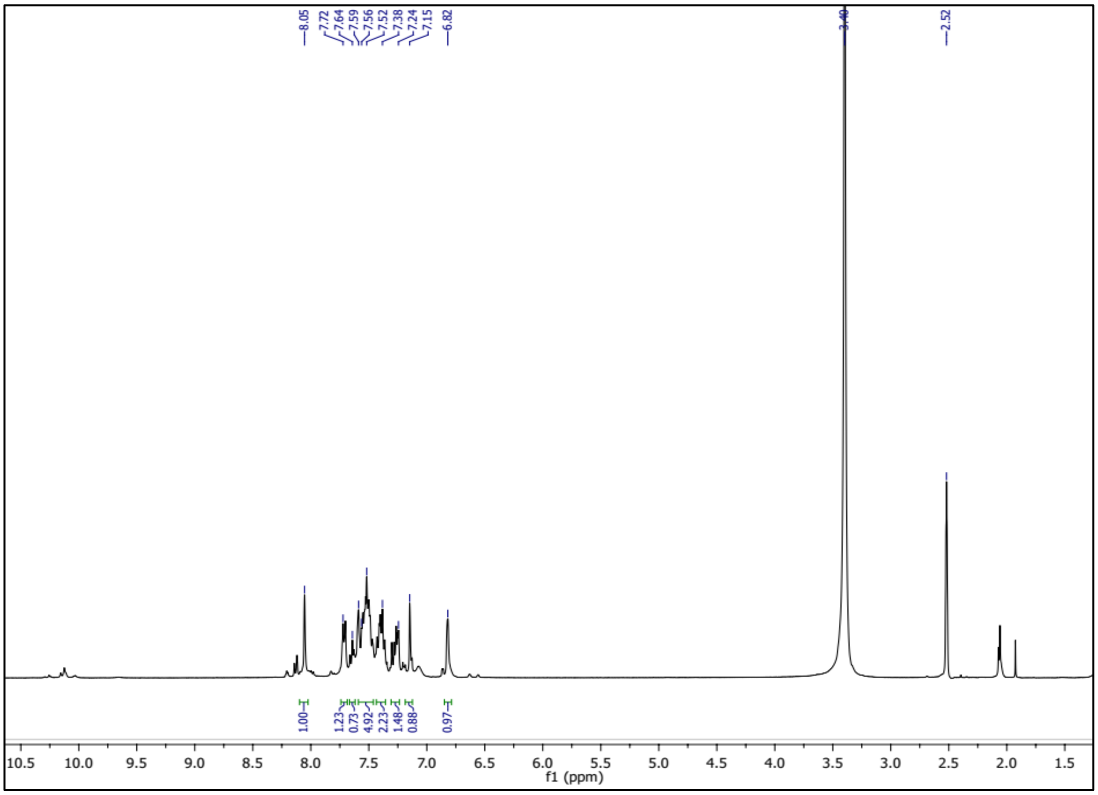

Figure S26. ^{1}HNMR chart of compound **6g**

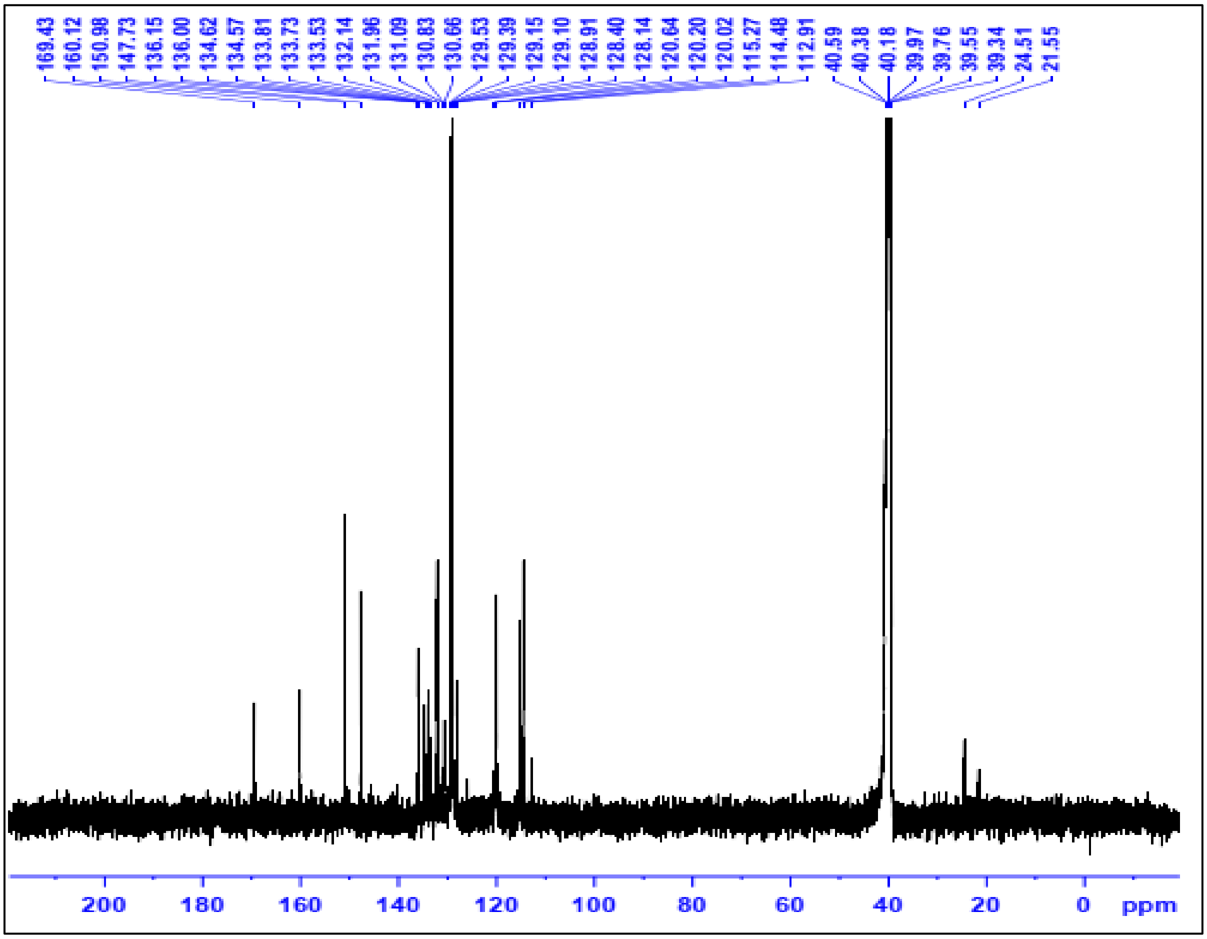

Figure S27. ^{13}CNMR chart of compound **6g**

3,3'-(diselanediylbis(4,1-phenylene))bis(2-phenyl-5-(thiophen-2-ylmethylene)-3,5-dihydro-4H-imidazol-4-one) (6h)

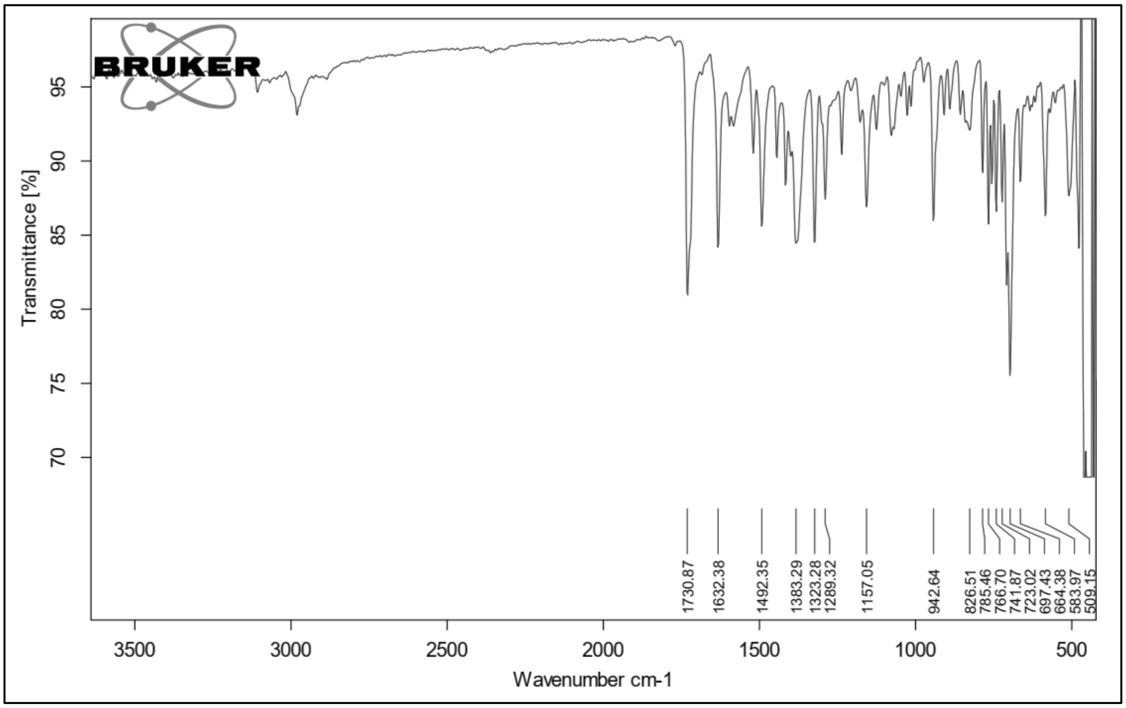

Figure S28. IR chart of compound **6h**

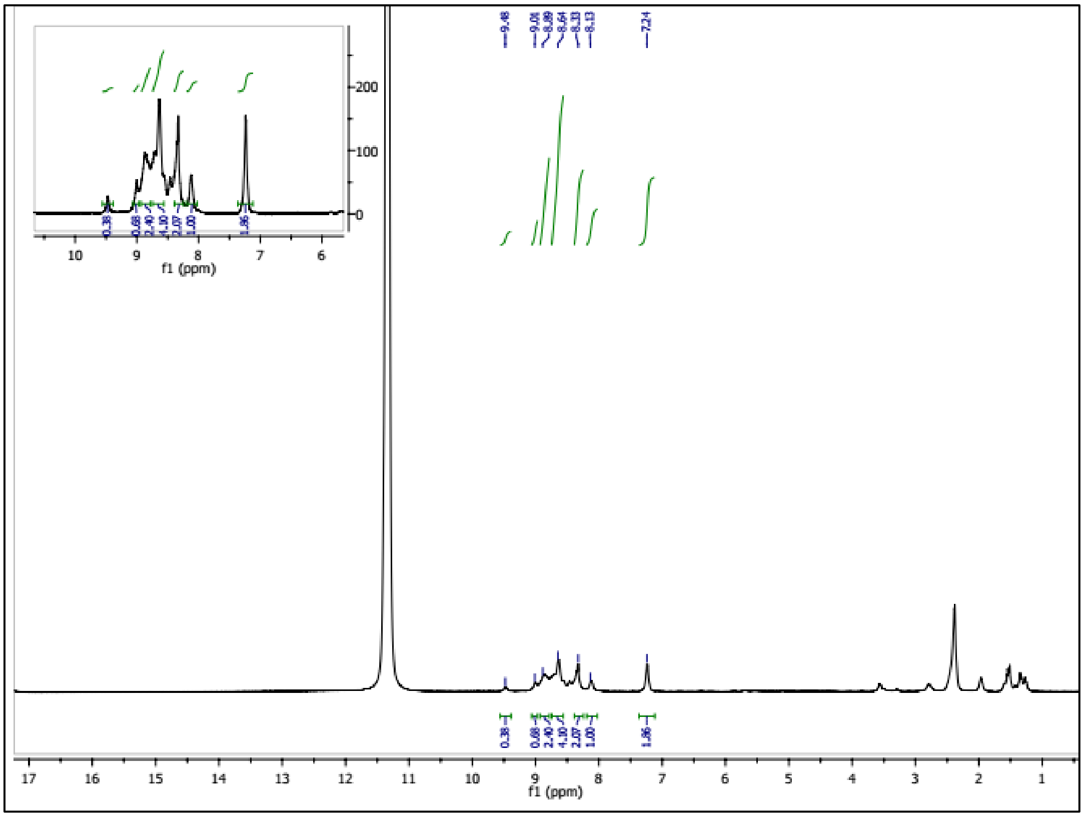

Figure S29. ^{1}HNMR chart of compound **6h**

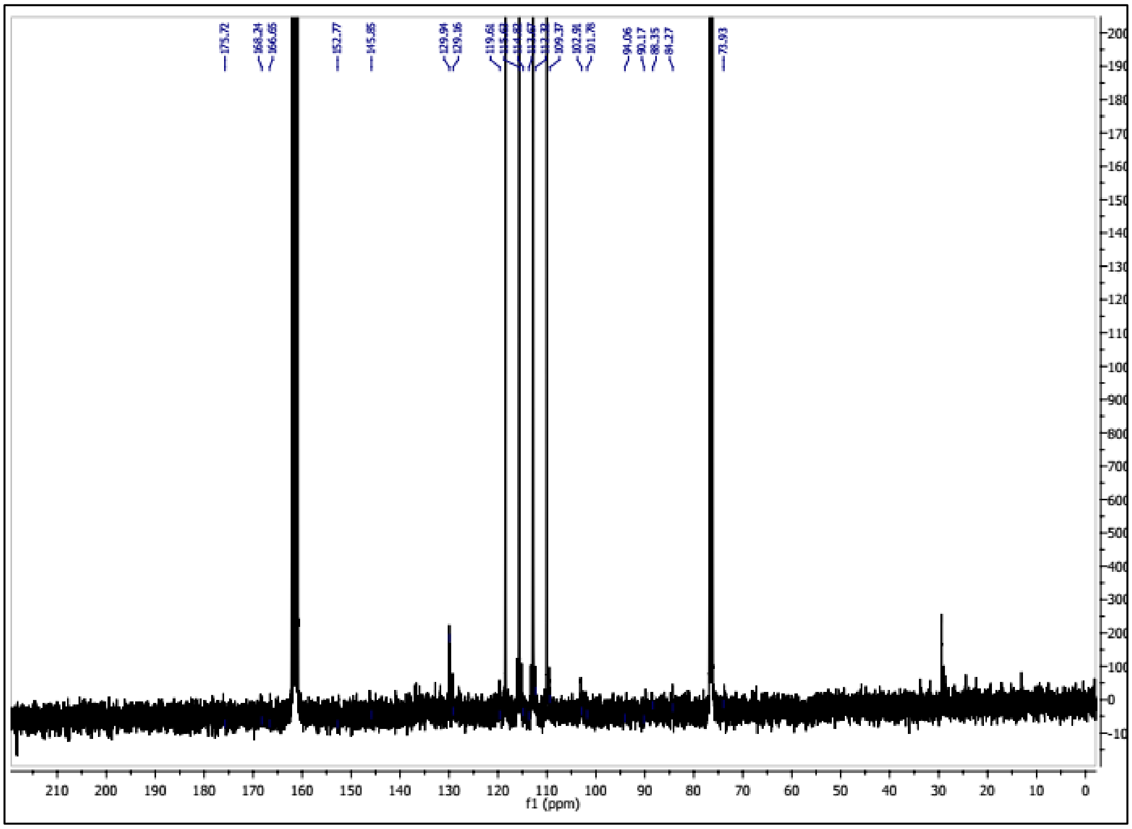

Figure S30. [13]NMR chart of compound **6h**

3,3'-(diselanediylbis(4,1-phenylene))bis(5-((10H-phenothiazin-2-yl)methylene)-2-phenyl-3,5-dihydro-4H-imidazol-4-one) (6i)

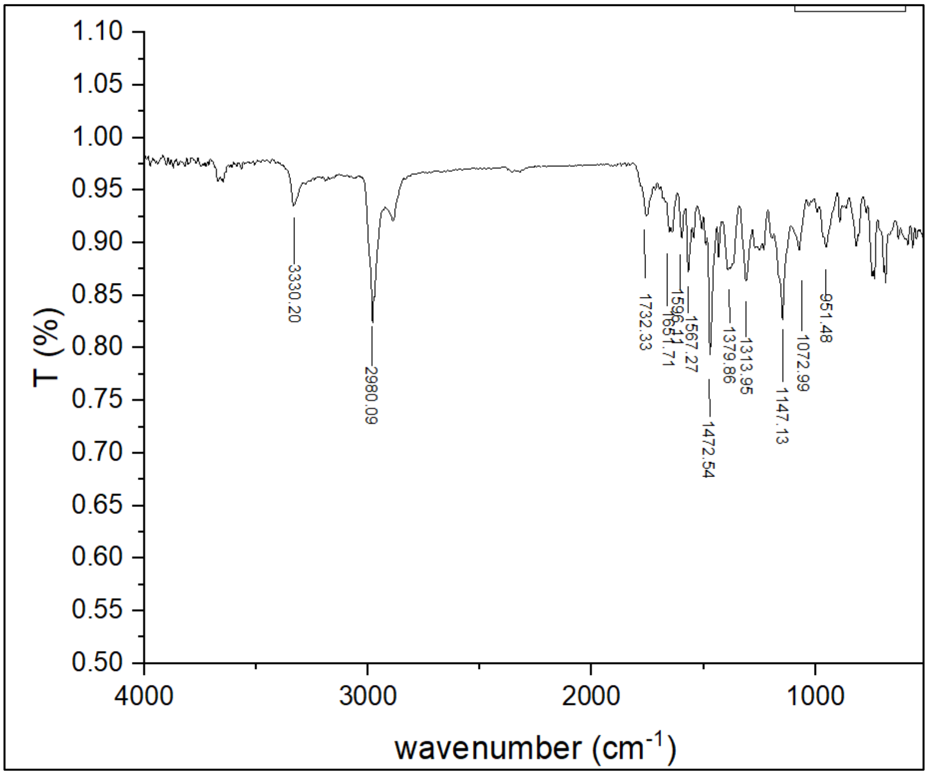

Figure S31. IR chart of compound **6i**

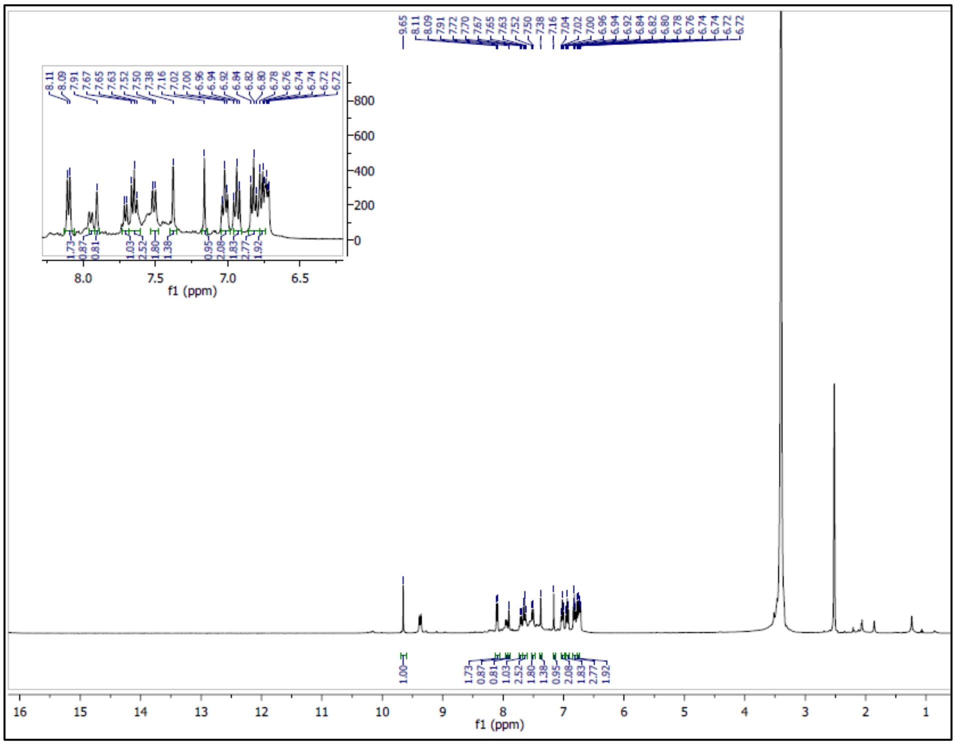

Figure S32. ^{1}HNMR chart of compound **6i**

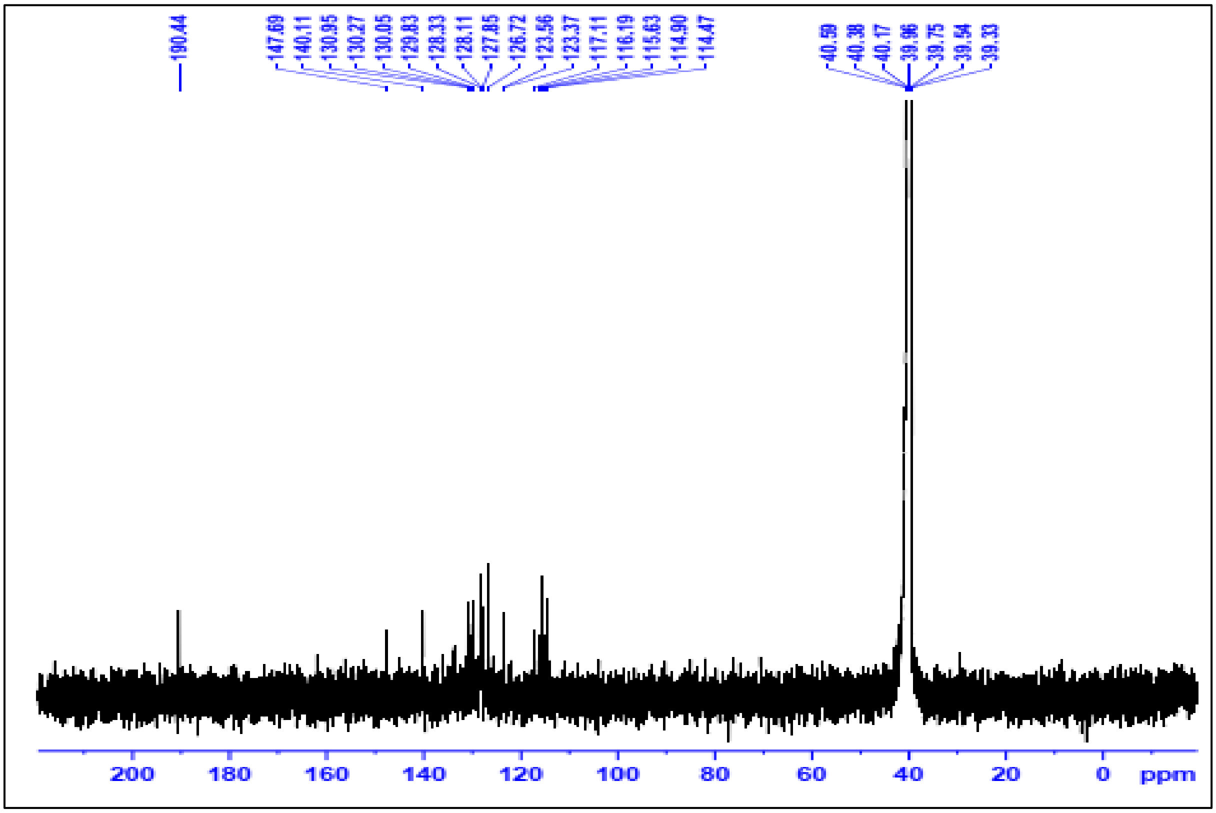

Figure S33. ^{13}CNMR chart of compound **6i**

3,3'-((diselanediylbis(4,1-phenylene))bis(5-oxo-2-phenyl-1,5-dihydro-4H-imidazole-1-yl-4-ylidene))bis(1-acetylindolin-2-one)(6j)

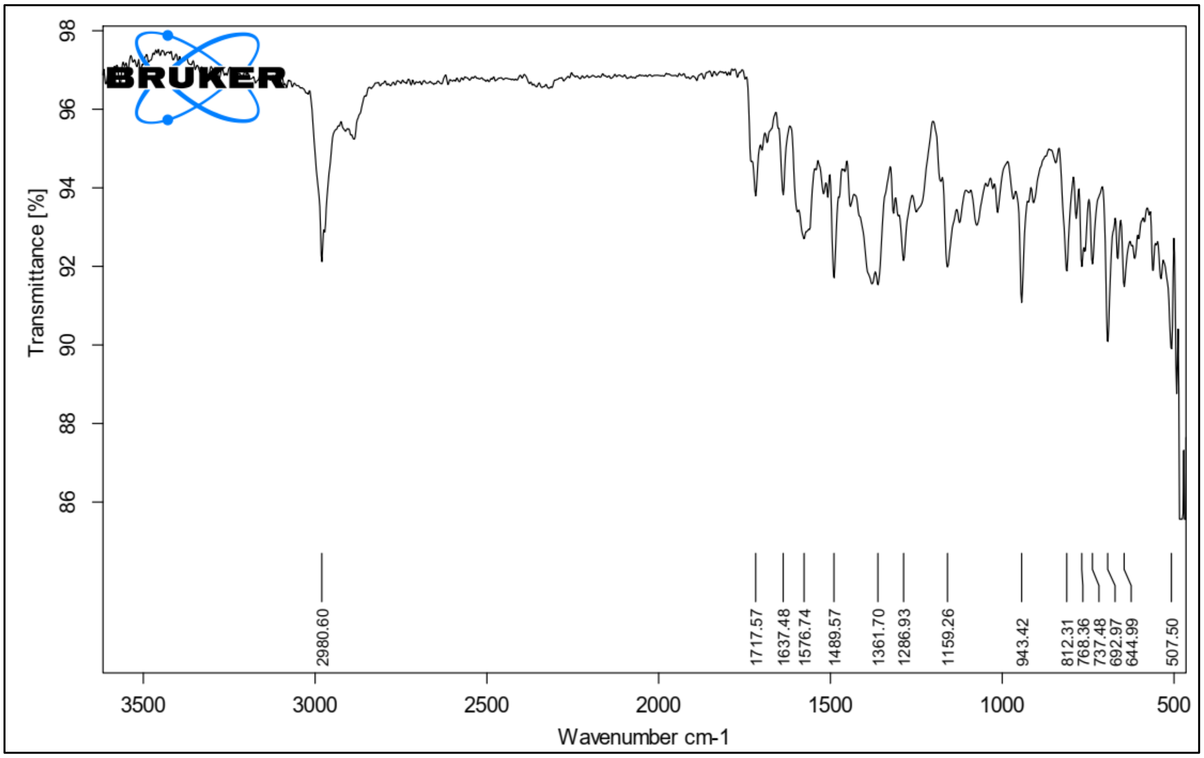

Figure S34. [1]R chart of compound **6j**

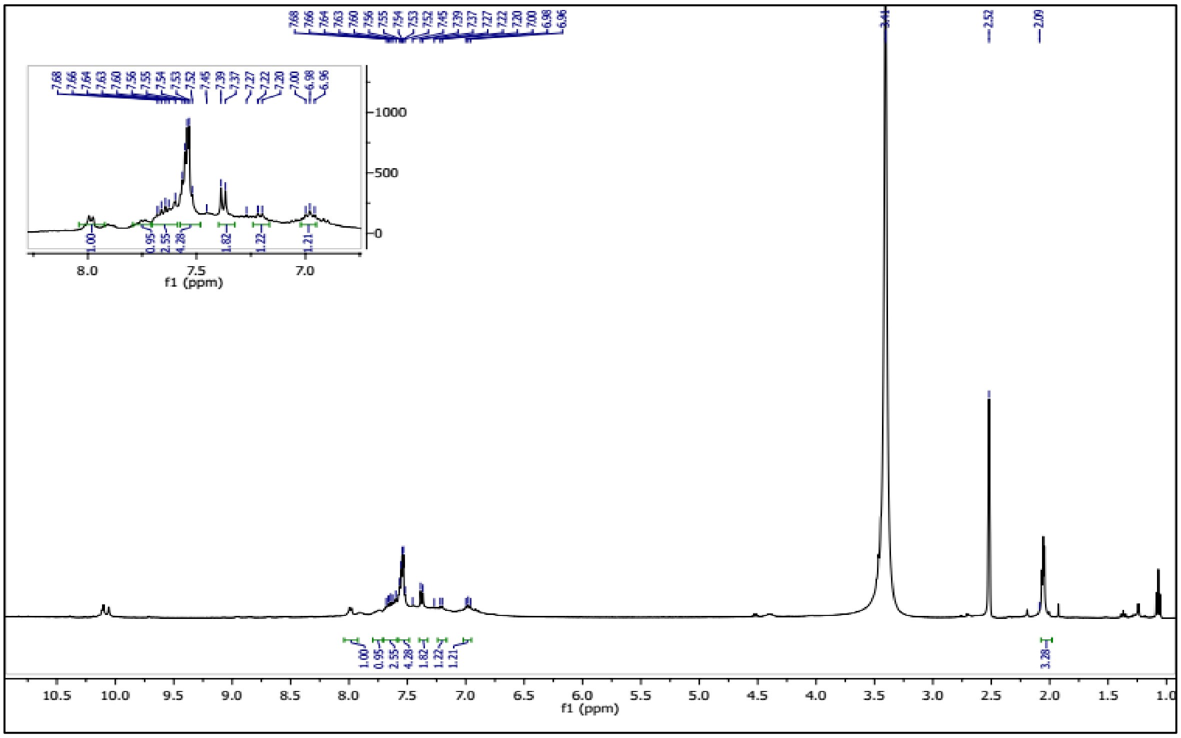

Figure S35. ^{1}HNMR chart of compound **6j**

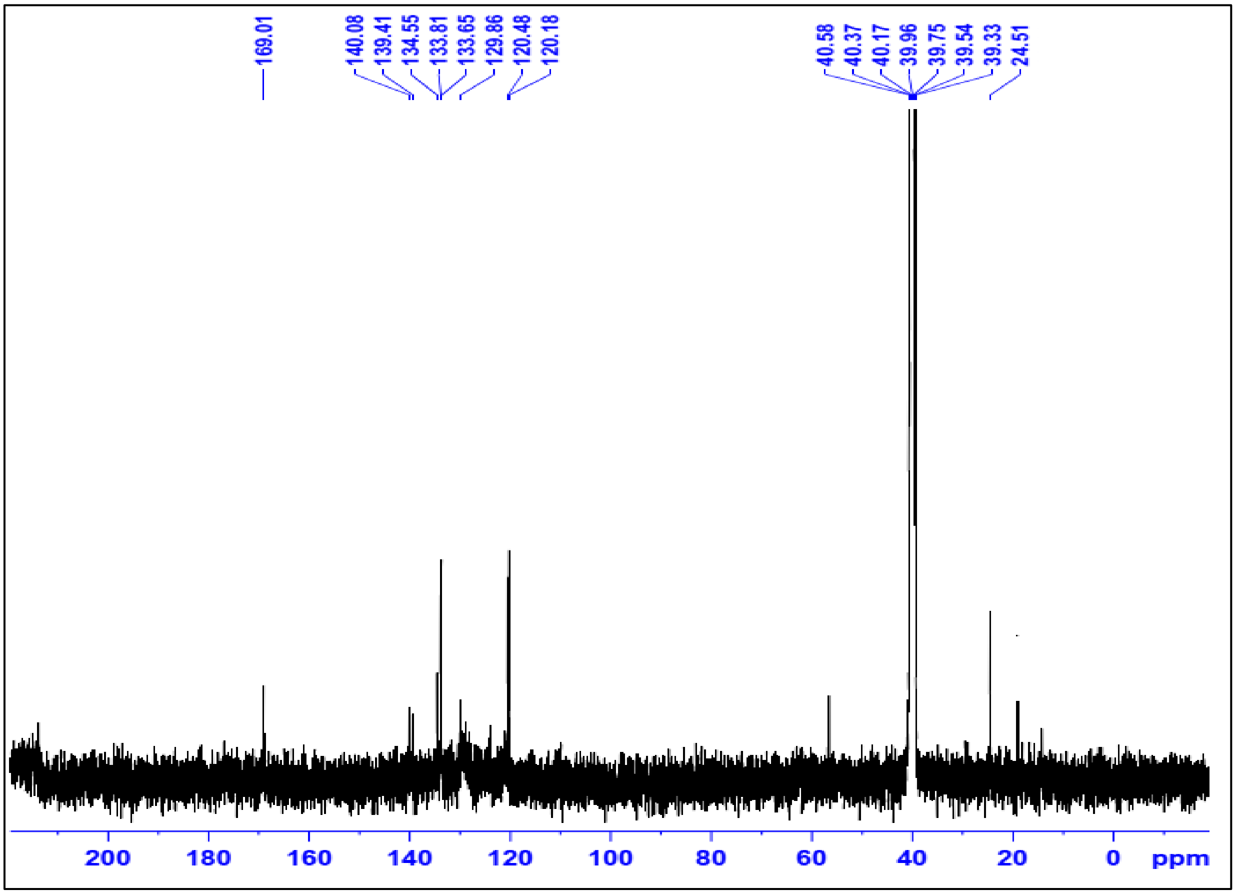

Figure S36. ^{13}CNMR chart of compound **6j**

Biological Data

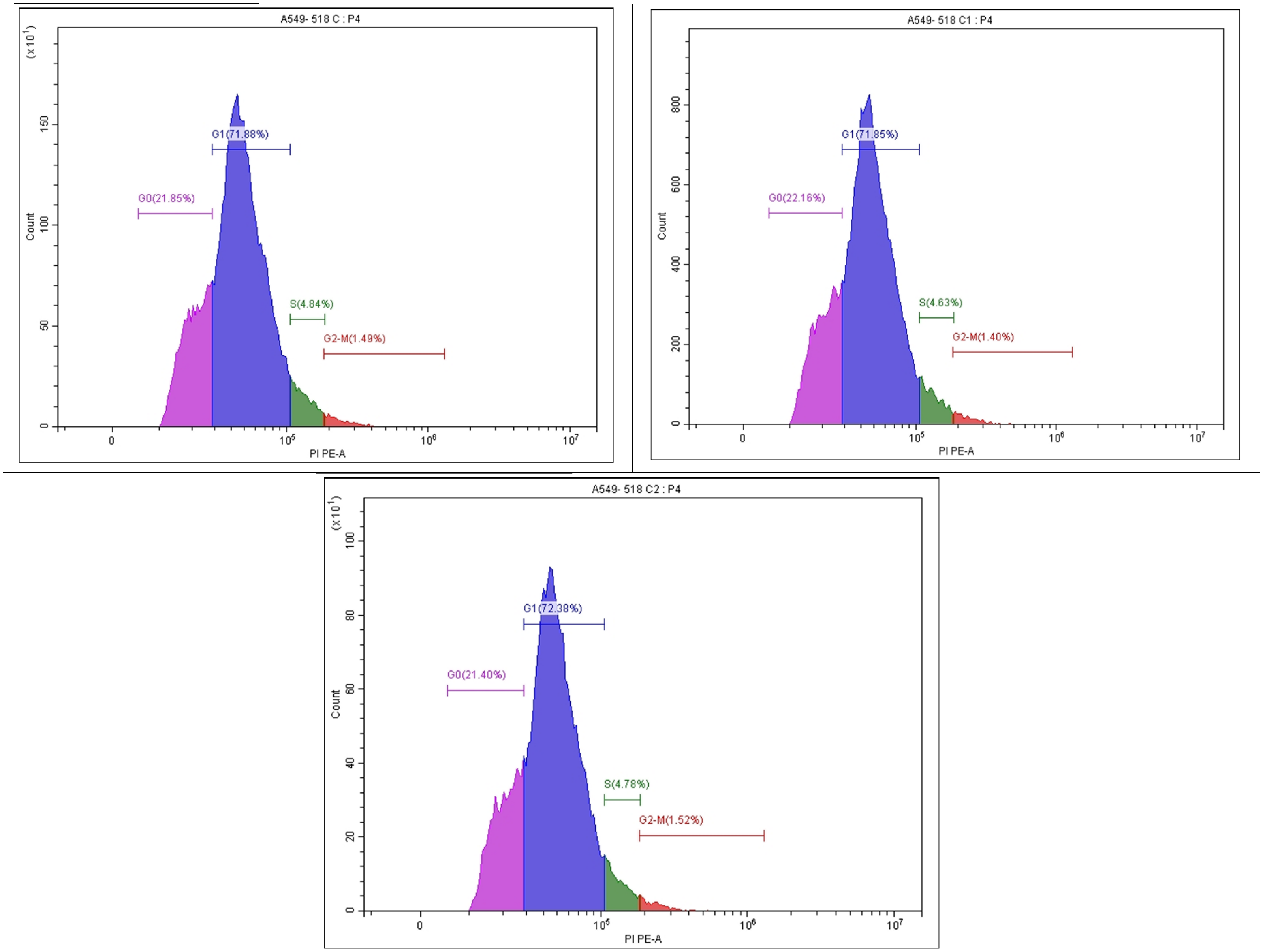

The triplicate experiments of the cell cycle analysis histogram of the untreated control A549 lung cancer cell line.

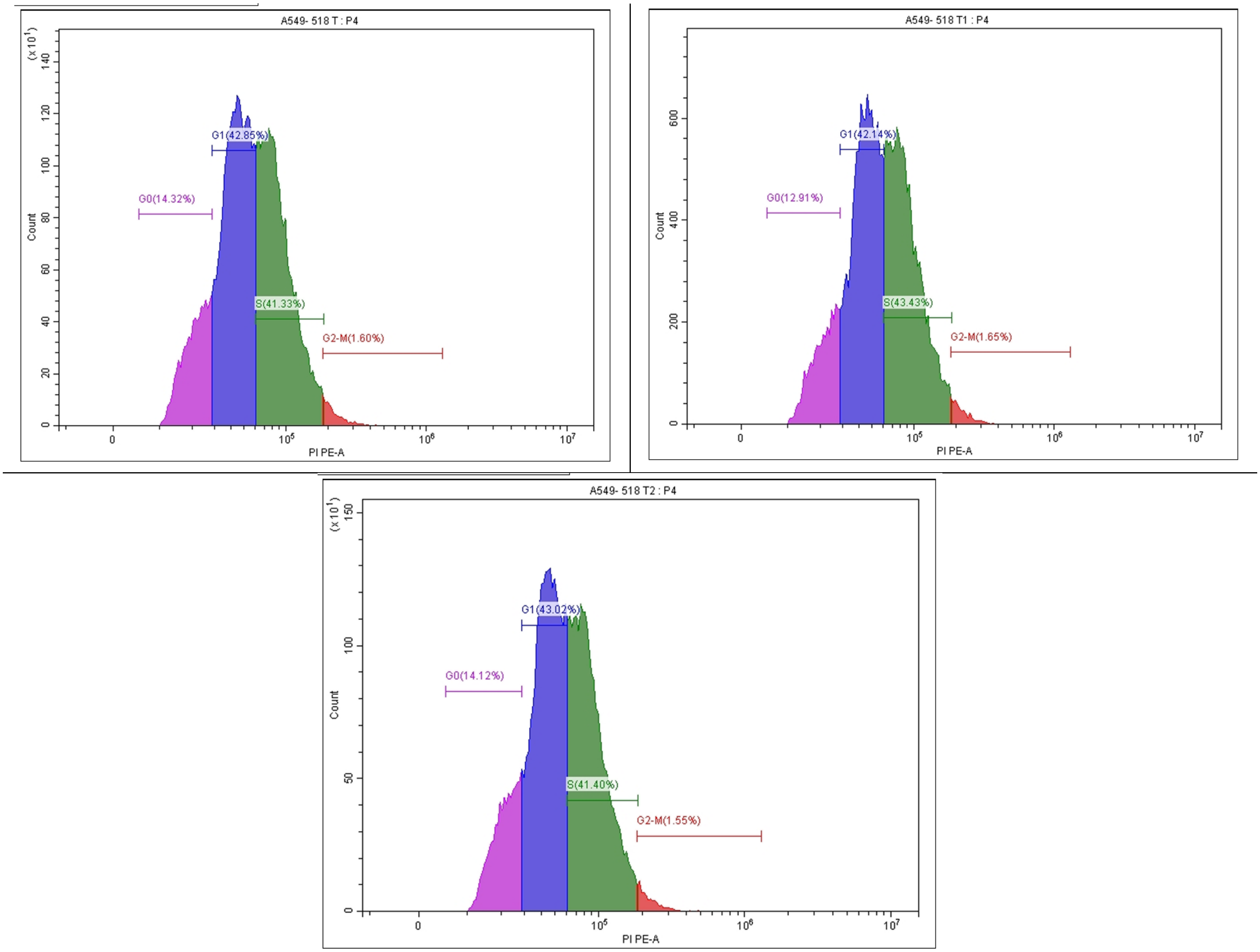

The triplicate experiments of the cell cycle analysis histogram of the **6g**-treated A549 lung cancer cell line.

Growth Inhibition% against human diverse cancer cell lines

The antitumor activities of the synthesized analogues (**6a-j**) against the selected 6 cancer cell lines were evaluated by sulphorhodamine-B (SRB) assay [5]. Briefly, cells were seeded at a density of 3×10^3 cells/well in 96-well microtiter plates. They were left to attach for 24 h before incubation with the aforementioned compounds. Next, cells were treated with 100 µg/mL for **6a-j** candidates.

For each concentration, three wells were used, and incubation was continued for 48 h. DMSO was used as a control vehicle (1 % v/v). At the end of incubation, cells were fixed with 20% trichloroacetic acid and stained with 0.4% SRB dye. The optical density (O.D.) of each well was measured spectrophotometrically at 570 nm using an ELISA microplate reader (TECAN sunrise™, Germany). The mean survival fraction at each drug concentration was calculated as follows: O.D. of the treated cells/O.D. of the control cells. The IC_{50} (concentration that produces 50% of cell growth inhibition) value of each drug was calculated using sigmoidal dose-response curve-fitting models (Graph Pad Prism software, version 8).

Cytotoxic inhibitory concentration 50 (IC_{50}) evaluation against PC3, MCF7, A549, and HCT_{116} cancer cell lines

The antitumor activities of the anticancer candidates (**6a-j**) against PC3, MCF7, A549, and HCT_{116} cancer cells were evaluated by sulphorhodamine-B (SRB) assay [5]. Briefly, cells were seeded at a density of 3×10^3 cells/well in 96-well microtiter plates. They were left to attach for 24 h before incubation with the aforementioned compounds. Next, cells were treated with different concentrations of 6.25, 12.5, 25, and 50 µg/mL for **6a-j** candidates.

For each concentration, three wells were used, and incubation was continued for 48 h. DMSO was used as a control vehicle (1 % *v/v*). At the end of incubation, cells were fixed with 20% trichloroacetic acid and stained with 0.4% SRB dye. The optical density (O.D.) of each well was measured spectrophotometrically at 570 nm using an ELISA microplate reader (TECAN sunrise™, Germany). The mean survival fraction at each drug concentration was calculated as follows: O.D. of the treated cells/O.D. of the control cells. The IC_{50} (concentration that produces 50% of cell growth inhibition) value of each drug was calculated using sigmoidal dose-response curve-fitting models (Graph Pad Prism software, version 8).

Assessment of apoptotic, anti-apoptotic, and antiangiogenic markers (Enzyme-linked Immunosorbent assay)

The microplate provided in this kit has been pre-coated with an antibody specific to CDK2, CDK4, CDK6, caspase-3, caspase-8, caspase-9, and VEGFR-2. Standards or samples are then added to the appropriate microplate wells with a biotin-conjugated antibody specific to CDK2, CDK4, CDK6, caspase-3, caspase-8, caspase-9, and VEGFR-2. Next, Avidin conjugated to Horseradish Peroxidase (HRP) is added to each microplate well and incubated. After the TMB substrate solution is added, only those wells that contain CDK2, CDK4, CDK6, caspase-3, caspase-8, caspase-9, and VEGFR-2, biotin-conjugated antibody, and enzyme-conjugated Avidin will exhibit a colour change. The enzyme-substrate reaction is terminated by the addition of sulphuric acid solution, and the colour change is measured spectrophotometrically at a wavelength of 450 nm ± 10 nm. The concentration of CDK2, CDK4, CDK6, caspase-3, caspase-8, caspase-9, and VEGFR-2 in the samples is then determined by comparing the O.D. of the samples to the standard curve. Average the duplicate readings for each standard, control, and sample, and subtract the average zero standard optical density. Construct a standard curve by plotting the mean O.D. and concentration for each standard and draw a best-fit curve through the points on the graph, or create a standard curve on log-log graph paper with CDK2, CDK4, CDK6, caspase-3, caspase-8, caspase-9, and VEGFR-2 concentration on the y-axis and absorbance on the x-axis. Using some plot software, for instance, Curve Expert 1.30, is also recommended. If samples have been diluted, the concentration read from the standard curve must be multiplied by the dilution factor.

Assay procedure:

1. Determine wells for diluted standard, blank, and analogue (**6g**). Prepare 7 wells for standard, 1 well for blank. Add 100 μL each of dilutions of standard, blank, and samples into the appropriate wells. Cover with the Plate sealer. Incubate for 1 h at 37 ^{o}C.
2. Remove the liquid from each well; don't wash.
3. Add 100 μL of Detection Reagent A working solution to each well, cover the wells with the plate sealer, and incubate for 1 h at 37 ^{o}C.
4. Aspirate the solution and wash with 350 μL of 1× Wash Solution to each well using a squirt bottle, multi-channel pipette, manifold dispenser, or auto washer, and let it sit for 1~2 min. Remove the remaining liquid from all wells completely by snapping the plate onto absorbent paper. Totally

wash 3 times. After the last wash, remove any remaining Wash Buffer by aspirating or decanting. Invert the plate and blot it against absorbent paper.

5. Add 100 µL of Detection Reagent B working solution to each well, cover the wells with the plate sealer, and incubate for 30 min at 37 ºC.

6. Repeat the aspiration/wash process for a total of 5 times as conducted in step 4.

7. Add 90 µL of Substrate Solution to each well. Cover with a new Plate sealer. Incubate for 10-20 min at 37 ºC (Don't exceed 30 min). Protect from light. The liquid will turn blue with the addition of a Substrate Solution.

8. Add 50 µL of Stop Solution to each well. The liquid will turn yellow with the addition of the stop solution. Mix the liquid by tapping the side of the plate. If the colour change does not appear uniform, gently tap the plate to ensure thorough mixing.

9. Remove any drops of water and fingerprints on the bottom of the plate and confirm there are no bubbles on the surface of the liquid. Then, run the microplate reader and conduct measurement at 450 nm immediately.

References

1. S. Shaaban, A. Negm, M. A. Sobh and L. A. Wessjohann, *European journal of medicinal chemistry*, 2015, **97**, 190-201.
2. M. Rostami, A. R. Khosropour, V. Mirkhani, I. Mohammadpoor-Baltork, M. Moghadam and S. Tangestaninejad, *Monatshefte für Chemie-Chemical Monthly*, 2011, **142**, 1175-1180.
3. B. Zhou and W. Chen, *Journal of Chemistry*, 2013, **2013**, 280585.
4. P. Anandgaonker, G. Kulkarni, S. Gaikwad and A. Rajbhoj, *Chinese Journal of Catalysis*, 2014, **35**, 196-200.
5. P. Skehan, R. Storeng, D. Scudiero, A. Monks, J. McMahon, D. Vistica, J. T. Warren, H. Bokesch, S. Kenney and M. R. Boyd, *JNCI: Journal of the National Cancer Institute*, 1990, **82**, 1107-1112.
